# Author Correction: Clonal architecture in mesothelioma is prognostic and shapes the tumour microenvironment

**DOI:** 10.1038/s41467-021-23867-6

**Published:** 2021-06-07

**Authors:** Min Zhang, Jin-Li Luo, Qianqian Sun, James Harber, Alan G. Dawson, Apostolos Nakas, Sara Busacca, Annabel J. Sharkey, David Waller, Michael T. Sheaff, Cathy Richards, Peter Wells-Jordan, Aarti Gaba, Charlotte Poile, Essa Y. Baitei, Aleksandra Bzura, Joanna Dzialo, Maymun Jama, John Le Quesne, Amrita Bajaj, Luke Martinson, Jacqui A. Shaw, Catrin Pritchard, Tamihiro Kamata, Nathaniel Kuse, Lee Brannan, Pan De Philip Zhang, Hongji Yang, Gareth Griffiths, Gareth Wilson, Charles Swanton, Frank Dudbridge, Edward J. Hollox, Dean A. Fennell

**Affiliations:** 1 Novogene Co., Ltd, Building 301, Beijing, China; 2 Bioinformatics and Biostatistics Support Hub, University of Leicester, Leicester, UK; 3 Department of Genetics and Genome Biology, University of Leicester, Leicester, UK; 4 Department of Cardiothoracic Surgery, Glenfield Hospital, Leicester, UK; 5 University of Sheffield Teaching Hospitals, Leicester, UK; 6 Barts Health NHS Trust, The Royal London Hospital, London, UK; 7 Department of Pathology, Leicester Royal Infirmary, Infirmary Square, Leicester, Leicestershire UK; 8 Department of Genetics, King Faisal Specialist Hospital and Research Centre, Riyadh, Saudi Arabia; 9 Institute of Cancer Sciences, University of Glasgow, Garscube Estate, Bearsden, UK; 10 Department of Radiology, Glenfield Hospital, Leicester, UK; 11 Department of Health Sciences, University of Leicester, Leicester, UK; 12 Department of Informatics, University of Leicester, Leicester, UK; 13 Southampton Clinical Trials Unit, University of Southampton, Southampton, UK; 14 The Francis Crick Institute, London, UK

**Keywords:** Cancer genomics, Mesothelioma, Tumour heterogeneity, Cancer genomics

Correction to: *Nature Communications* 10.1038/s41467-021-21798-w, published online 19 March 2021.

The original version of this Article contained an error in the spelling of the author Luke Martinson, which was incorrectly given as Luke Martison.

This has now been corrected in both the PDF and HTML versions of the Article.

