# Supplementary material for: Clonal architecture in mesothelioma is prognostic and shapes the tumour microenvironment
Source: Nat Commun. 2021 Mar 19;12:1751. doi: 10.1038/s41467-021-21798-w (PMC7979861; doi:10.1038/s41467-021-21798-w)
Supplement: Supplementary file 1 — Supplementary Information [file 41467_2021_21798_MOESM1_ESM.pdf]

SUPPLEMENTARY FIGURES

Supplementary Figure 1

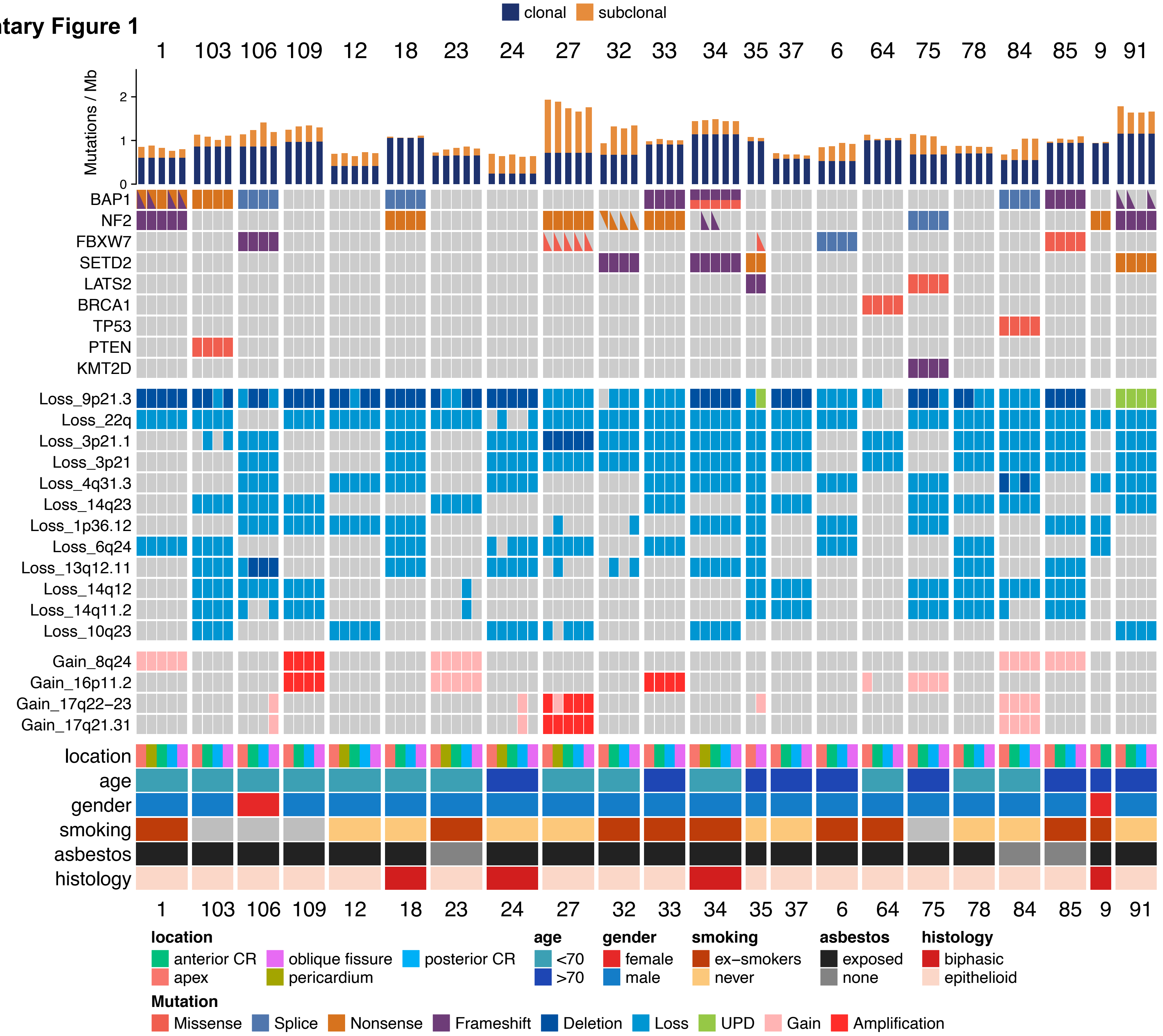

Supplementary Figure 1. Clonal versus subclonal driver alterations in MPM

Overview of somatic driver alterations, including single nucleotide variants (SNVs), insertion-deletions (INDELs) and somatic copy number alterations (SCNAs). In the second panel, the triangle indicates subclonal mutations. *BAP1* in MED001 and *NF2* in MED032 are evolved in parallel subclonally. Patients with unknown smoking history are colour coded as grey in the heatmap.

Supplementary Figure 2

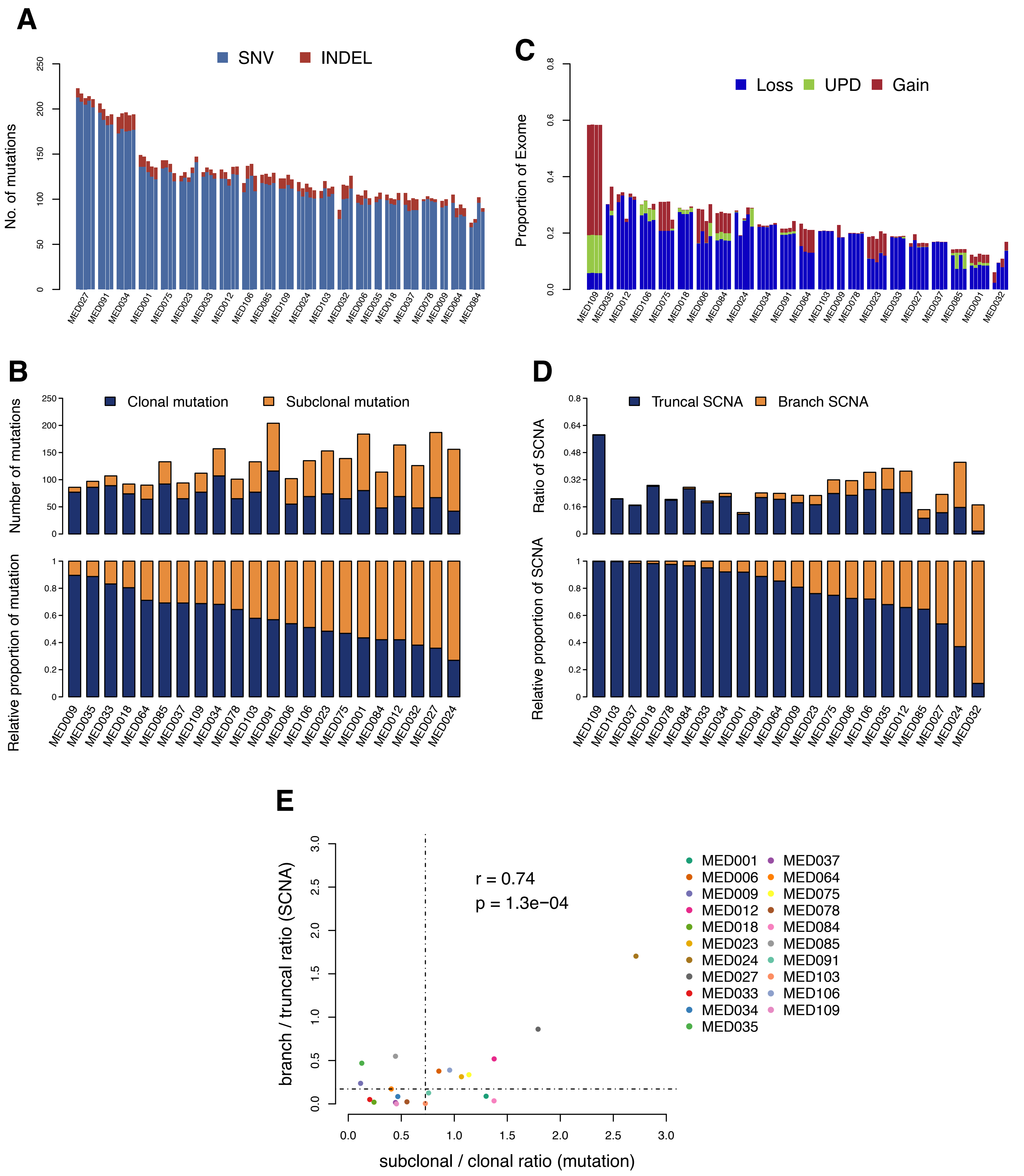

Supplementary Figure 2. Genomic Intratumour heterogeneity in MPM

Mutational burden and intratumour heterogeneity (ITH). Histogram showing the number of somatic SNV/INDELs (A) ranked by total number of mutations (high on the left to low on the right) (B) Histograms showing the proportion of mutations that are either clonal (dark blue) or subclonal (orange). Top histogram shows the absolute number of mutations, lower histograms show the proportion of mutations that are clonal versus subclonal. (C) Histogram showing the proportion of the genome affected by loss (blue), gain (red) or uniparental disomy (UPD, green) ranked by proportion of the genome affected (high on the left, to low on the right). (D) Histograms showing the proportion of somatic copy number alterations (SCNAs) that are either clonal (dark blue) or subclonal (orange). Top histogram shows the absolute number of SCNAs, lower histograms show the proportion of SCNAs that are clonal versus subclonal ranked by clonal proportion (high on the left to low on the right). (E) Graph showing the correlation between subclonal/clonal ratio for mutations (X-axis) versus SCNAs (y-axis) for the cohort. The correlation coefficient is denoted r, with associated p value denoted p.

Supplementary Figure 3

A

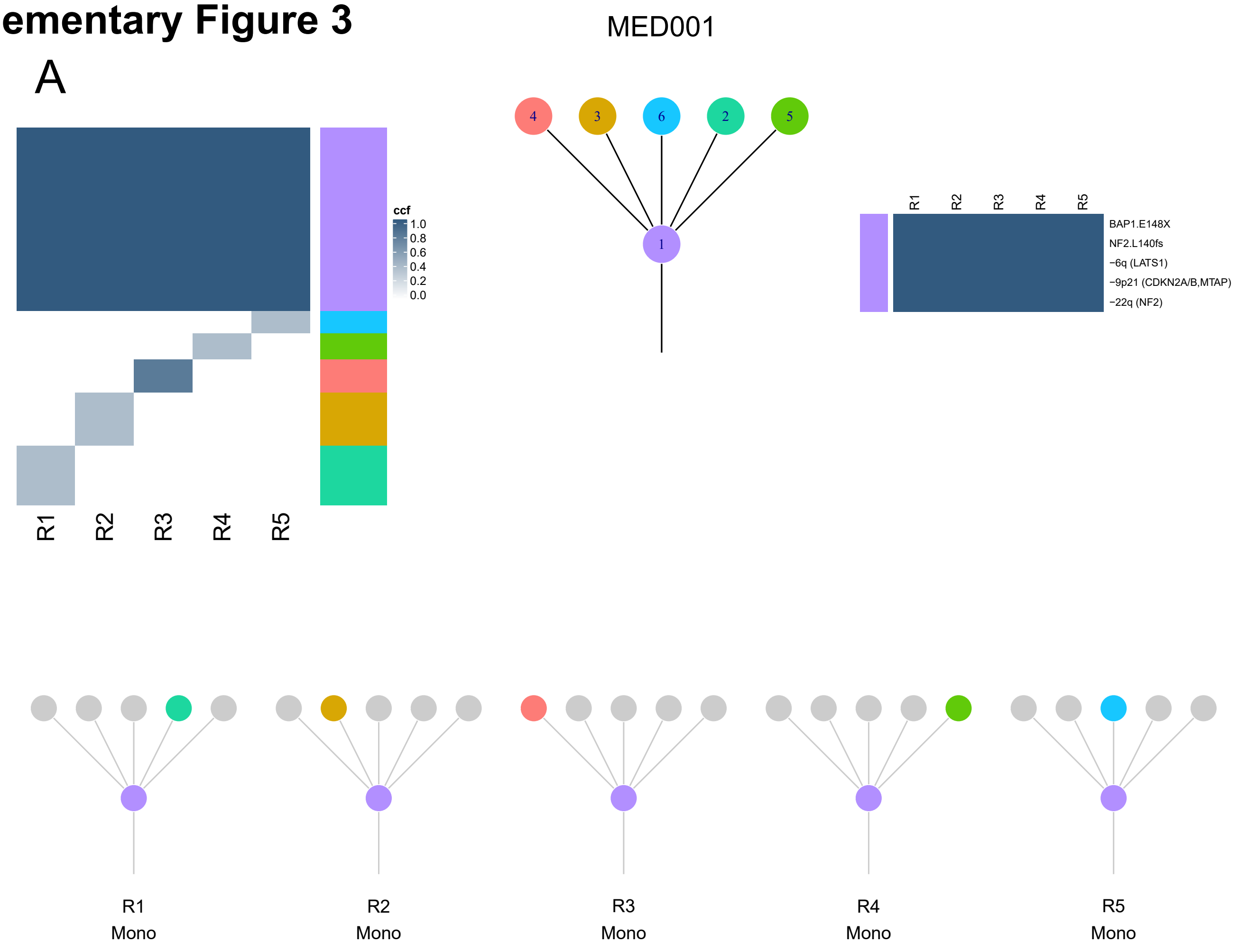

B

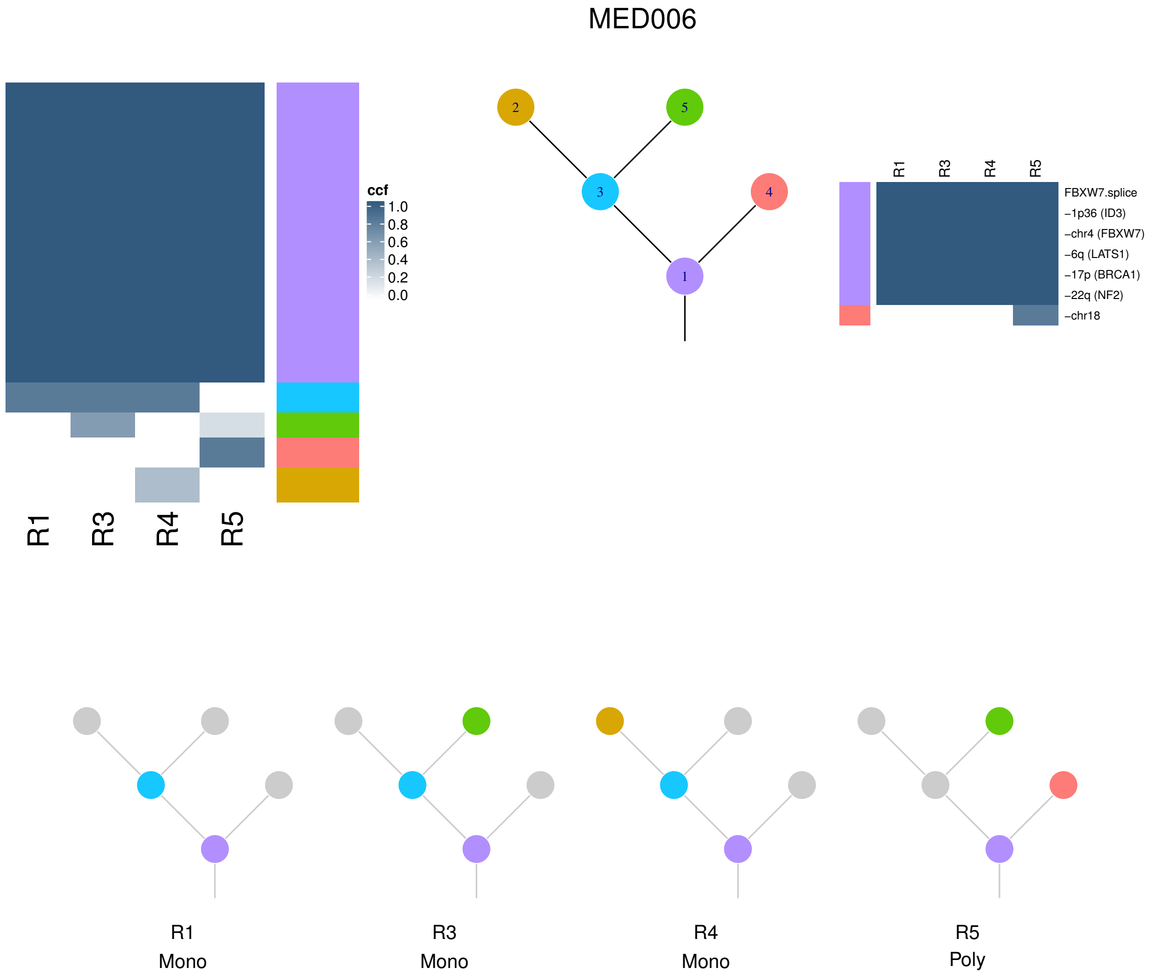

C

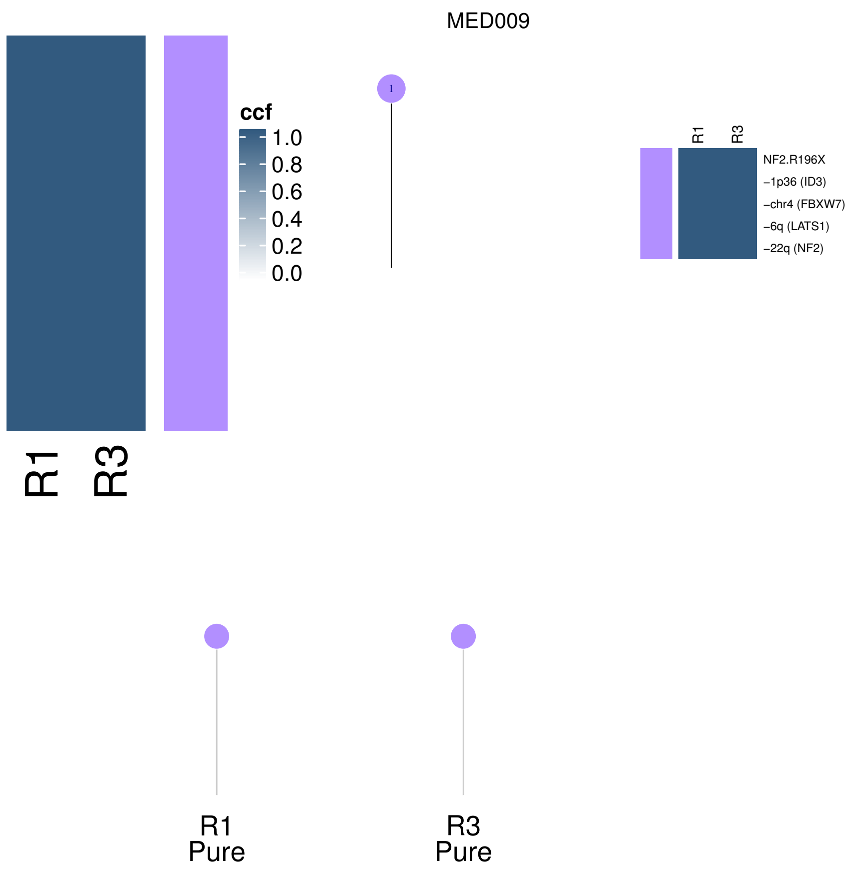
MED009
ccf
1.0
0.8
0.6
0.4
0.2
0.0
R1
R3
1
NF2.R196X
-1p36 (ID3)
-chr4 (FBXW7)
-6q (LATS1)
-22q (NF2)
R1
Pure
R3
Pure

D

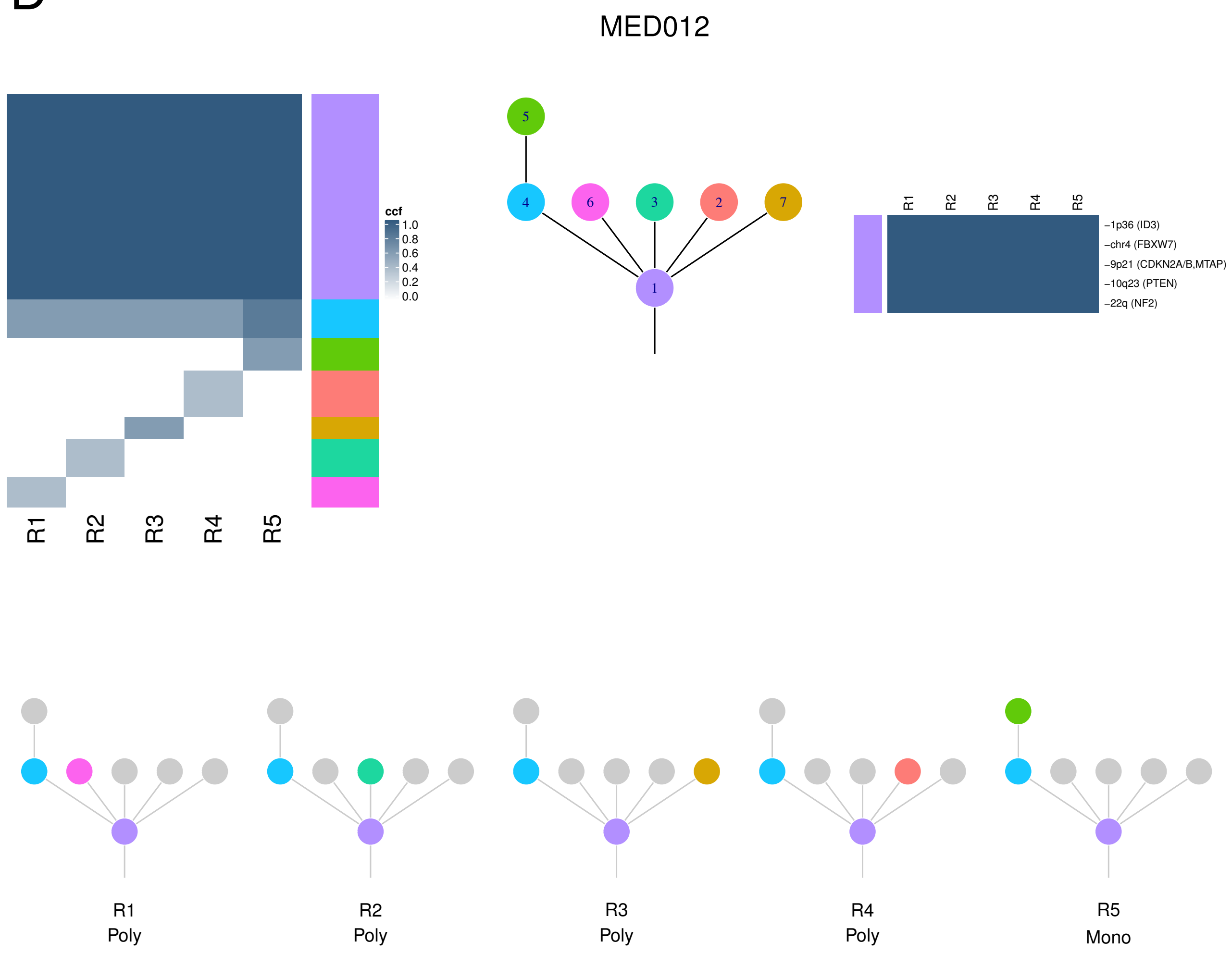
MED012
ccf
1.0
0.8
0.6
0.4
0.2
0.0
R1
R2
R3
R4
R5
5
4
6
3
2
7
1
-1p36 (ID3)
-chr4 (FBXW7)
-9p21 (CDKN2A/B,MTAP)
-10q23 (PTEN)
-22q (NF2)
R1
Poly
R2
Poly
R3
Poly
R4
Poly
R5
Mono

E

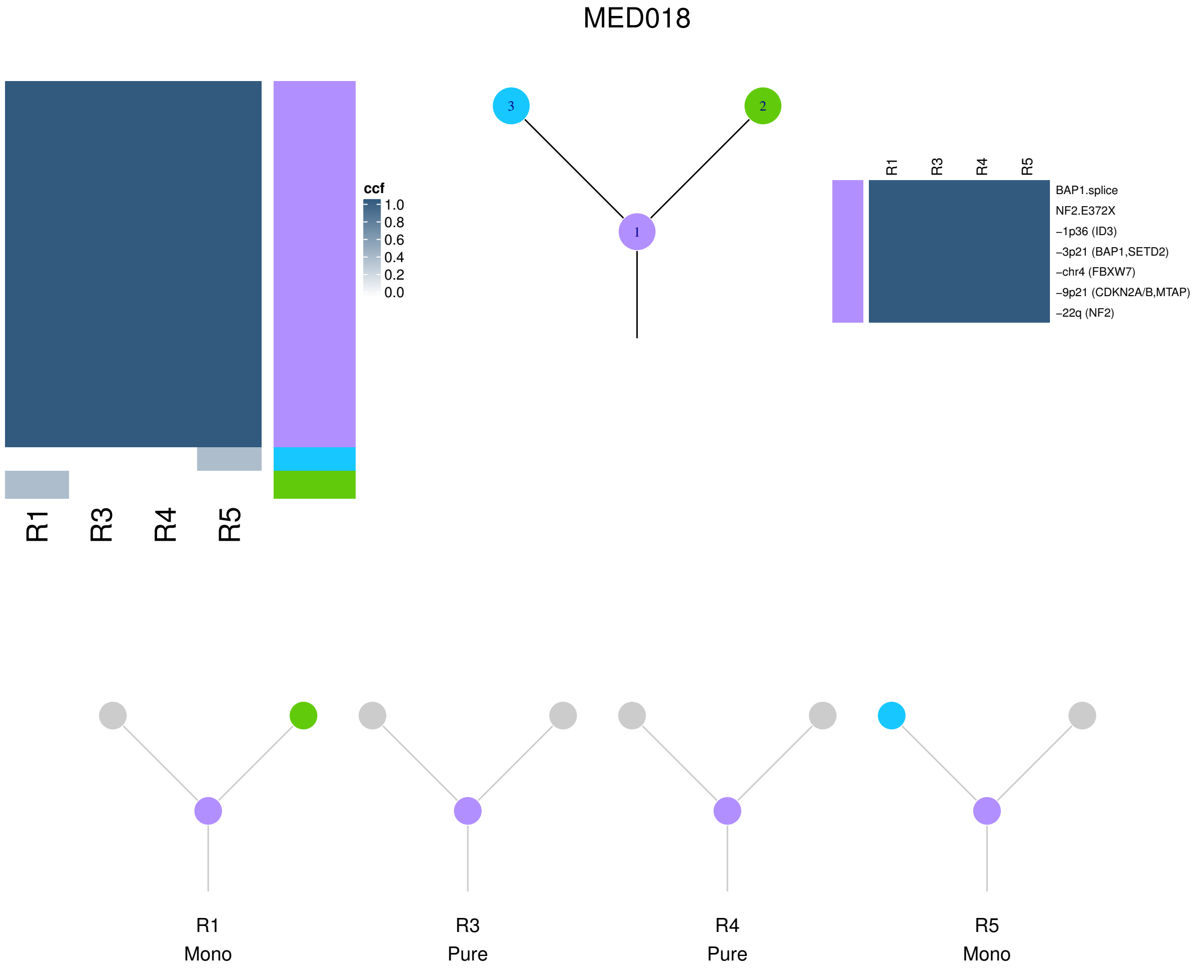

MED018
ccf
1.0
0.8
0.6
0.4
0.2
0.0
R1
R3
R4
R5
BAP1.splice
NF2.E372X
−1p36 (ID3)
−3p21 (BAP1,SETD2)
−chr4 (FBXW7)
−9p21 (CDKN2A/B,MTAP)
−22q (NF2)
R1 Mono
R3 Pure
R4 Pure
R5 Mono

F

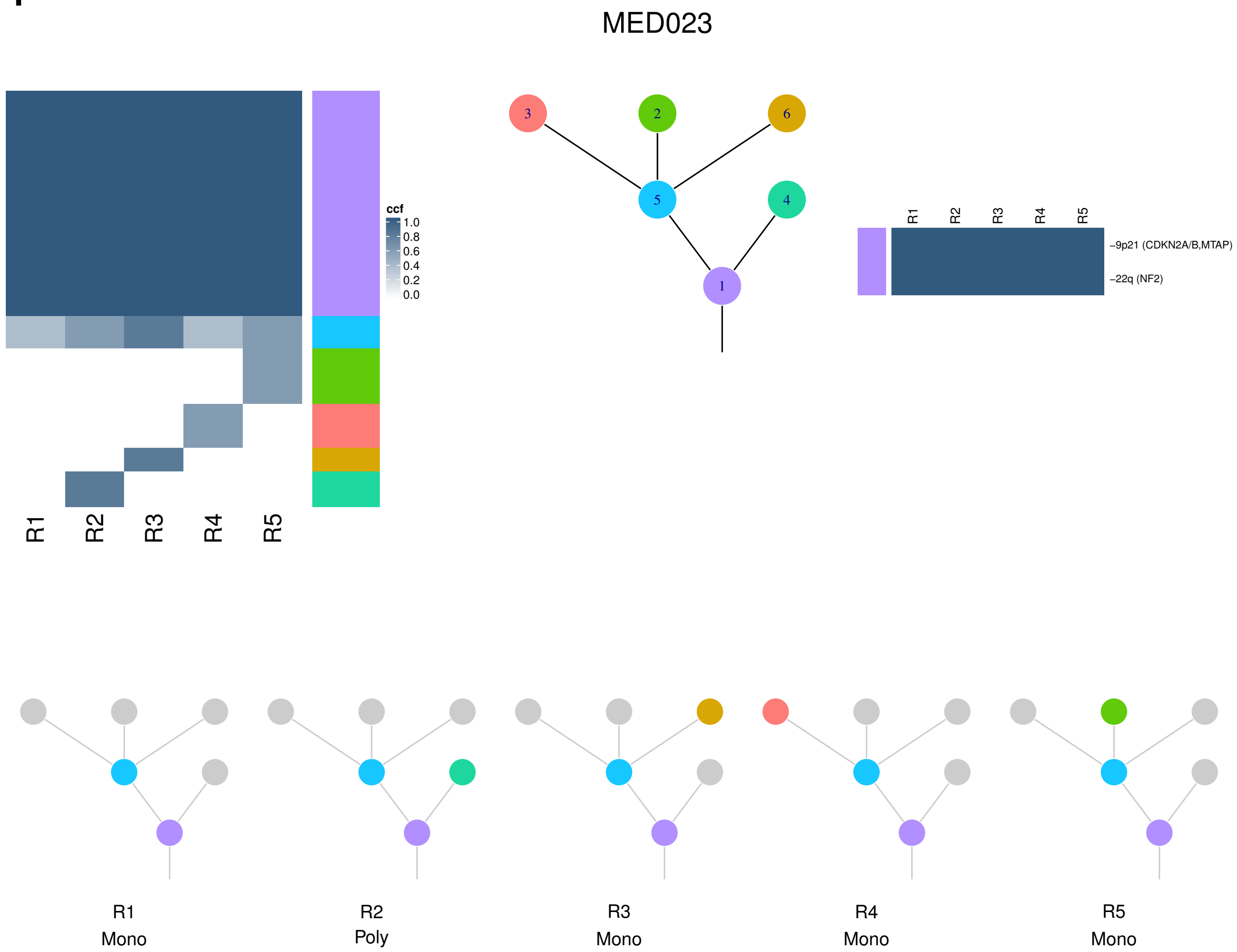

MED023
ccf
1.0
0.8
0.6
0.4
0.2
0.0
R1
R2
R3
R4
R5
−9p21 (CDKN2A/B,MTAP)
−22q (NF2)
R1 Mono
R2 Poly
R3 Mono
R4 Mono
R5 Mono

G

MED024

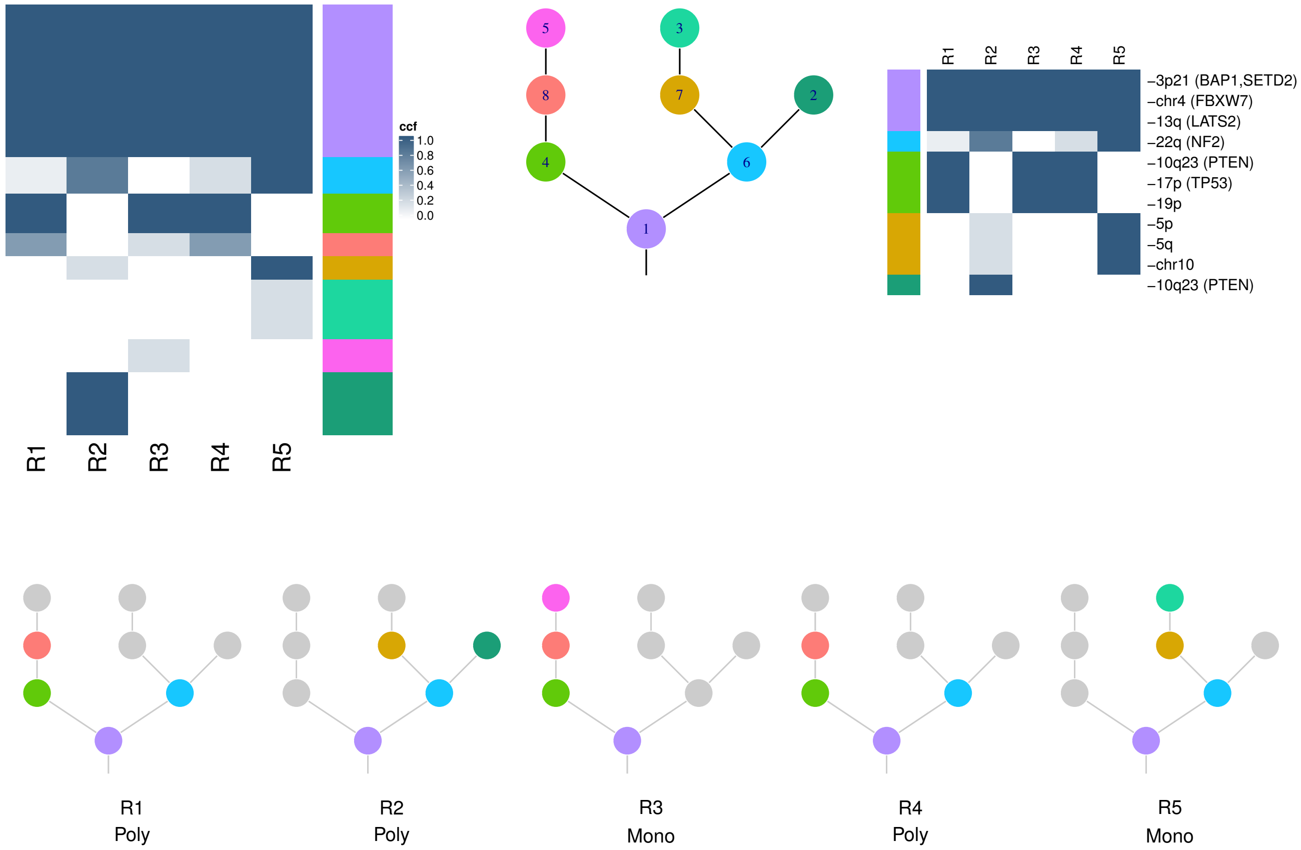

H

MED027

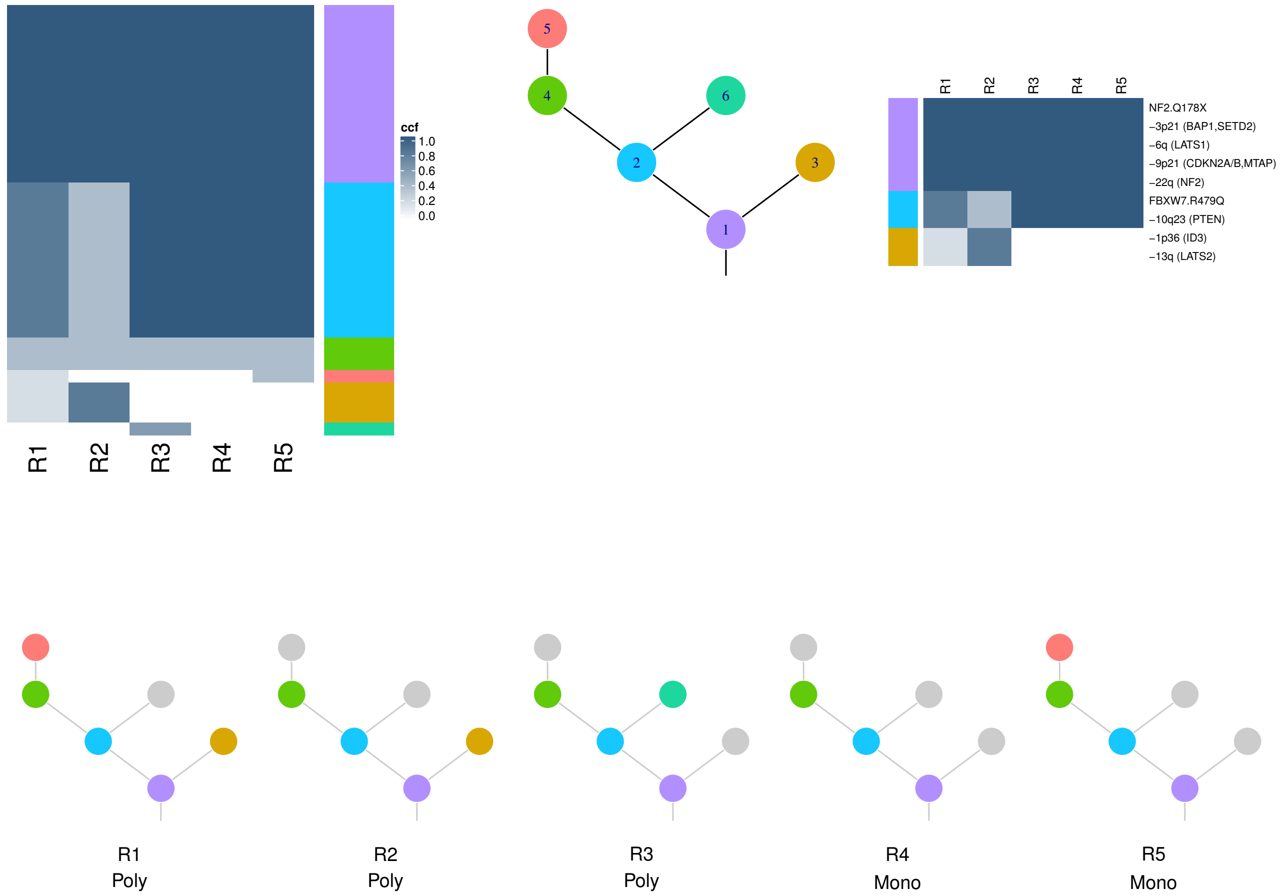

I

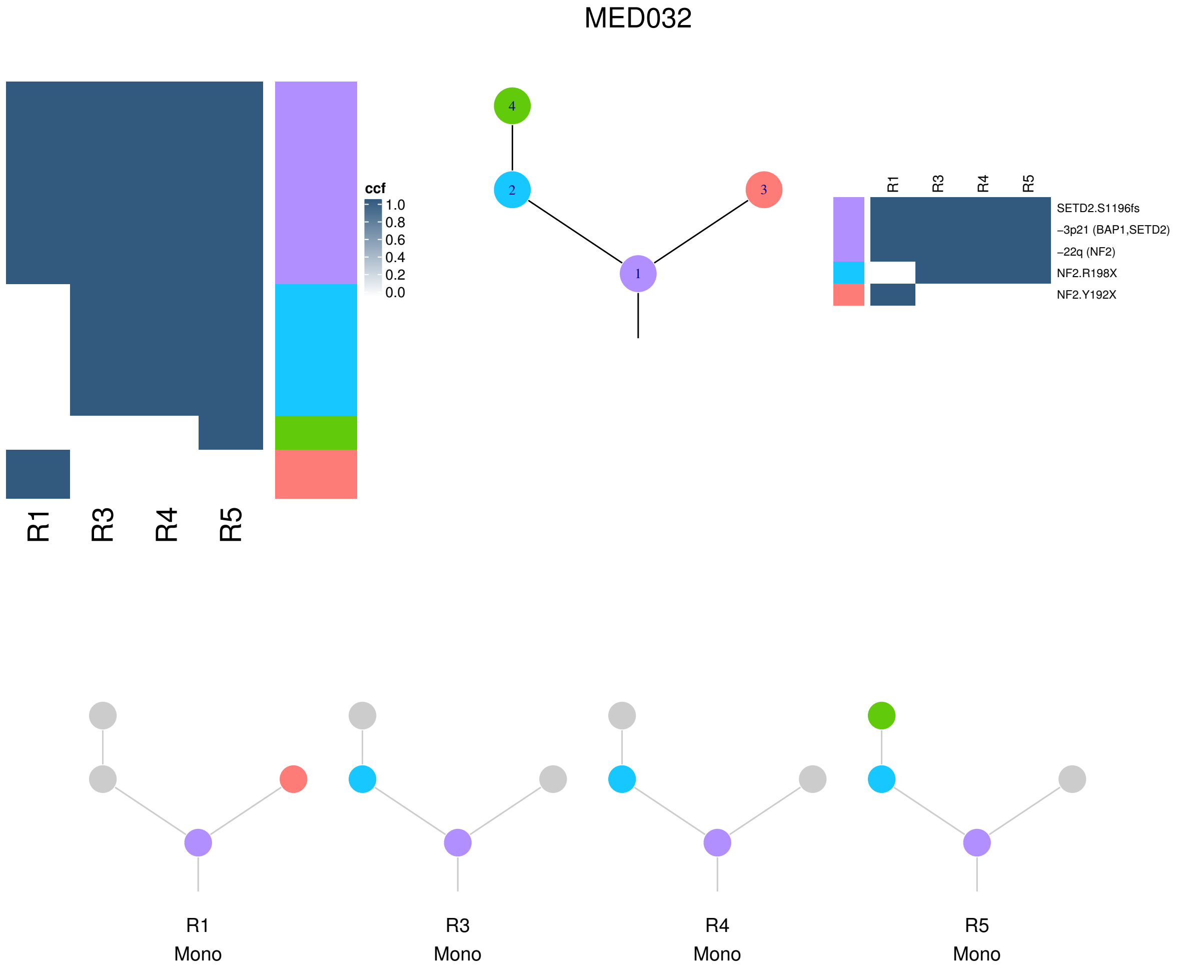
MED032
ccf
1.0
0.8
0.6
0.4
0.2
0.0
R1
R3
R4
R5
SETD2.S1196fs
−3p21 (BAP1,SETD2)
−22q (NF2)
NF2.R198X
NF2.Y192X
R1
Mono
R3
Mono
R4
Mono
R5
Mono

J

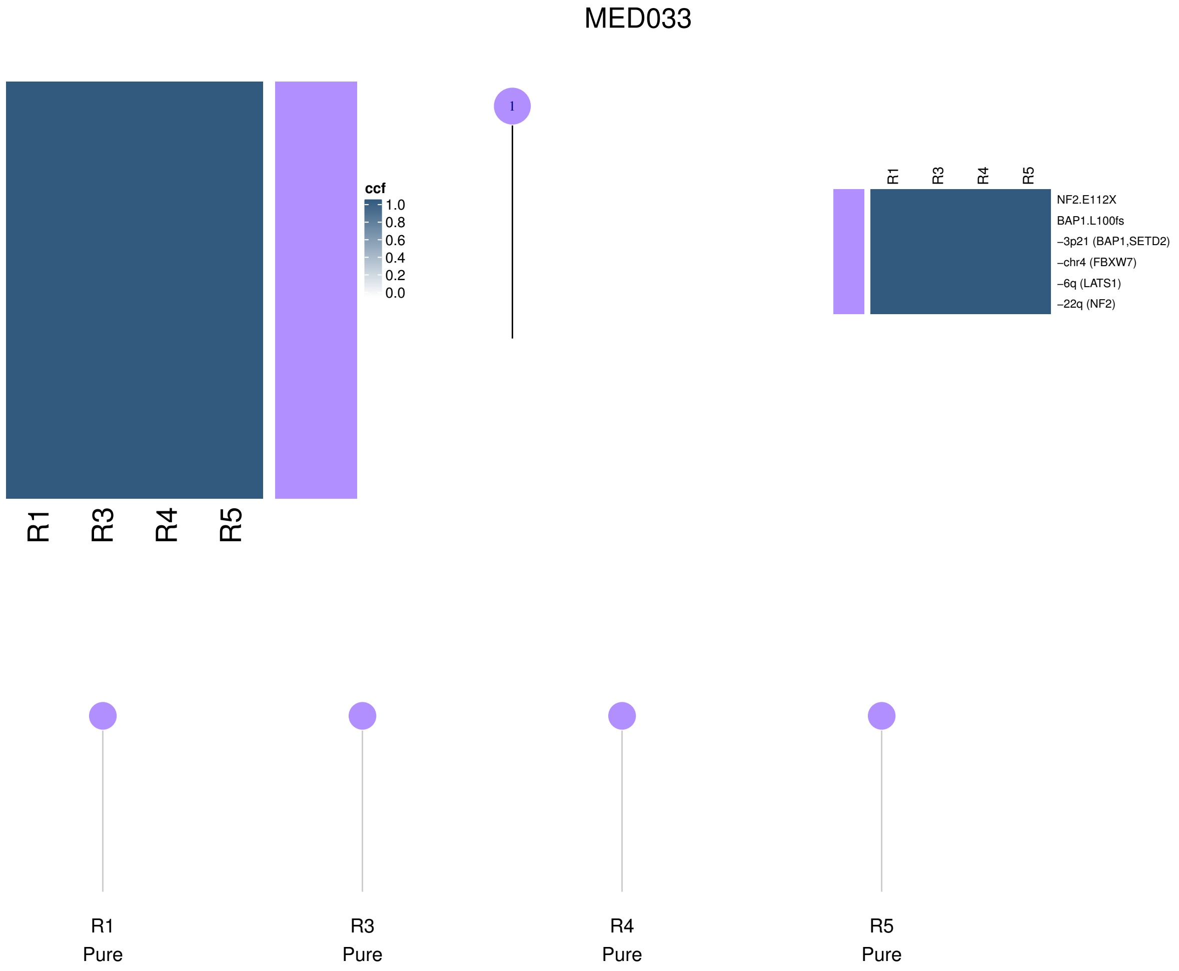
MED033
ccf
1.0
0.8
0.6
0.4
0.2
0.0
R1
R3
R4
R5
NF2.E112X
BAP1.L100fs
−3p21 (BAP1,SETD2)
−chr4 (FBXW7)
−6q (LATS1)
−22q (NF2)
R1
Pure
R3
Pure
R4
Pure
R5
Pure

K

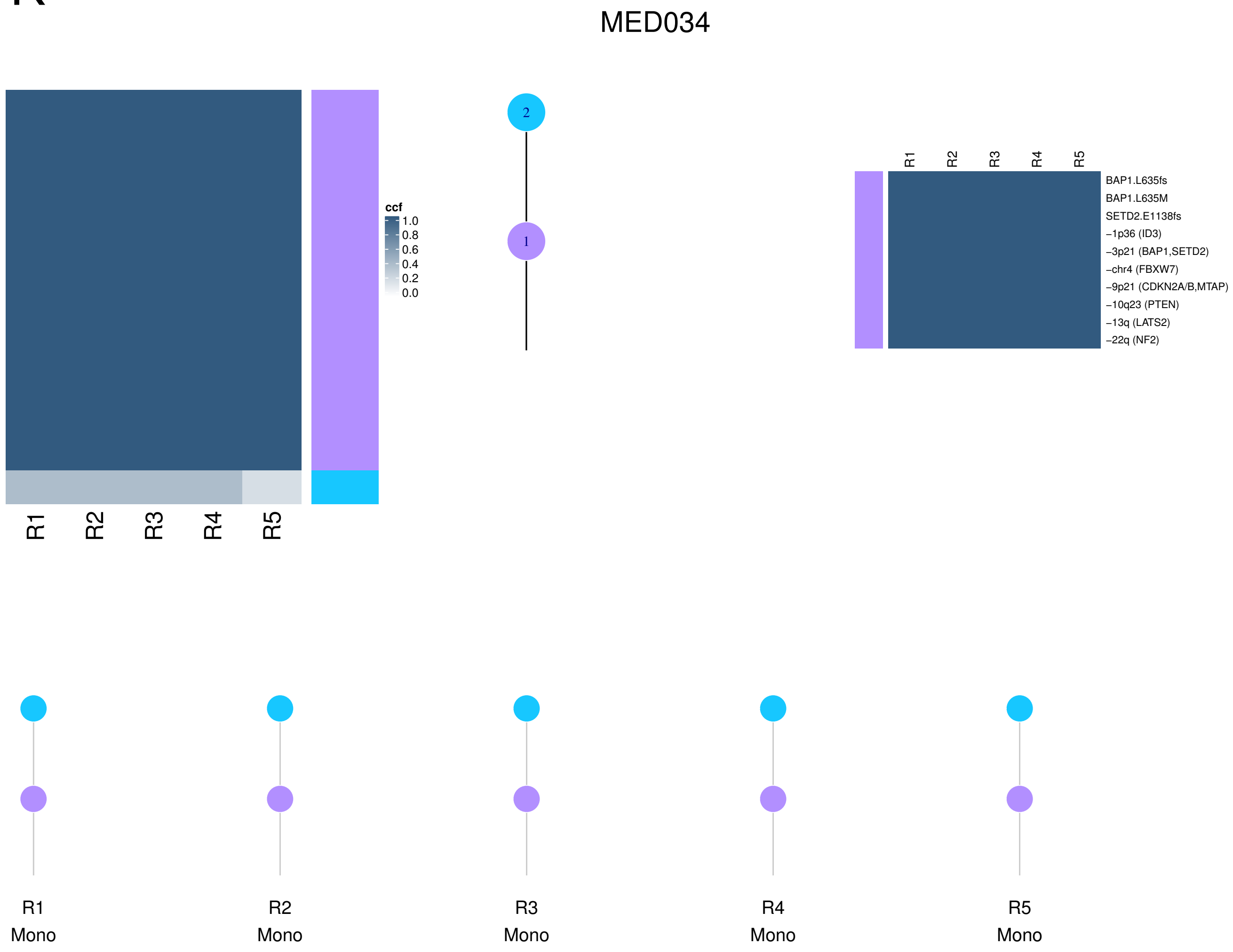
MED034
ccf
1.0
0.8
0.6
0.4
0.2
0.0
R1
R2
R3
R4
R5
2
1
BAP1.L635fs
BAP1.L635M
SETD2.E1138fs
−1p36 (ID3)
−3p21 (BAP1,SETD2)
−chr4 (FBXW7)
−9p21 (CDKN2A/B,MTAP)
−10q23 (PTEN)
−13q (LATS2)
−22q (NF2)
R1 Mono
R2 Mono
R3 Mono
R4 Mono
R5 Mono

L

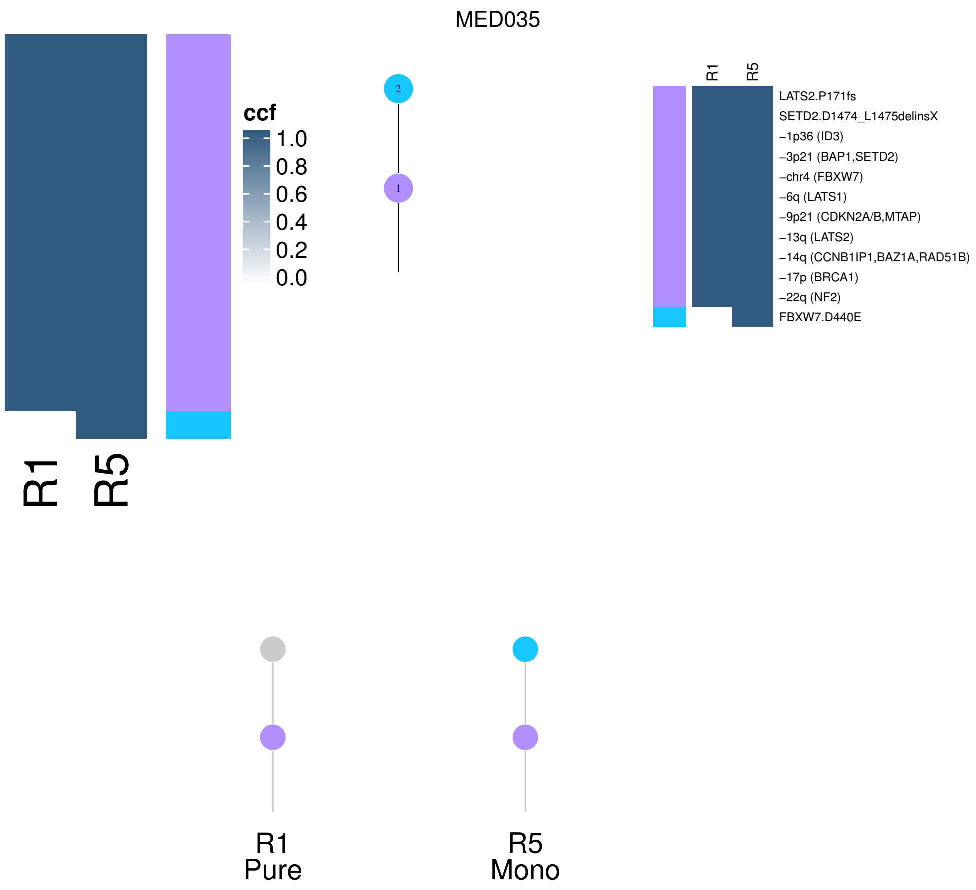
MED035
ccf
1.0
0.8
0.6
0.4
0.2
0.0
R1
R5
2
1
LATS2.P171fs
SETD2.D1474_L1475delinsX
−1p36 (ID3)
−3p21 (BAP1,SETD2)
−chr4 (FBXW7)
−6q (LATS1)
−9p21 (CDKN2A/B,MTAP)
−13q (LATS2)
−14q (CCNB1IP1,BAZ1A,RAD51B)
−17p (BRCA1)
−22q (NF2)
FBXW7.D440E
R1 Pure
R5 Mono

M

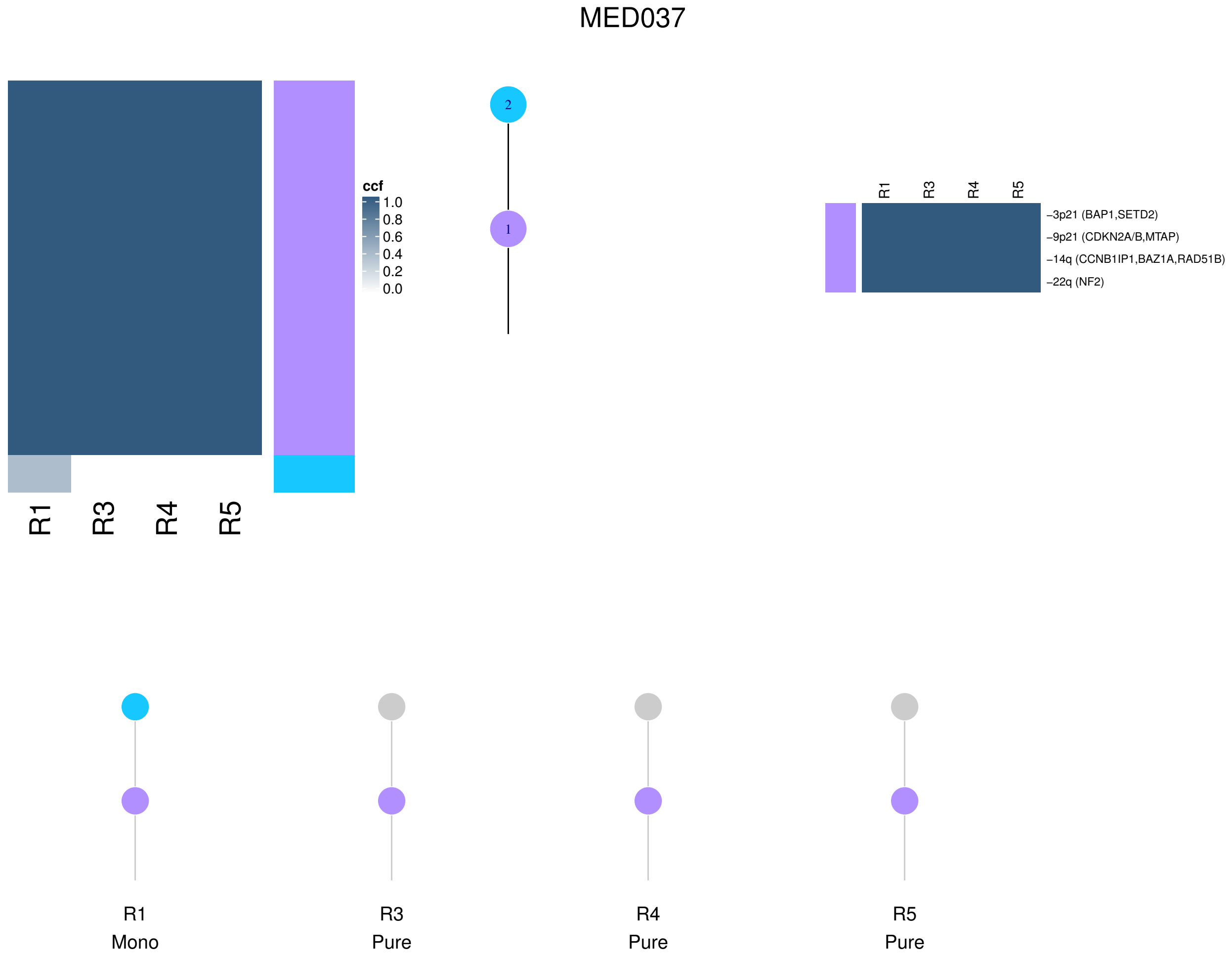

MED037
ccf
1.0
0.8
0.6
0.4
0.2
0.0
R1
R3
R4
R5
2
1
−3p21 (BAP1,SETD2)
−9p21 (CDKN2A/B,MTAP)
−14q (CCNB1IP1,BAZ1A,RAD51B)
−22q (NF2)
R1
Mono
R3
Pure
R4
Pure
R5
Pure

N

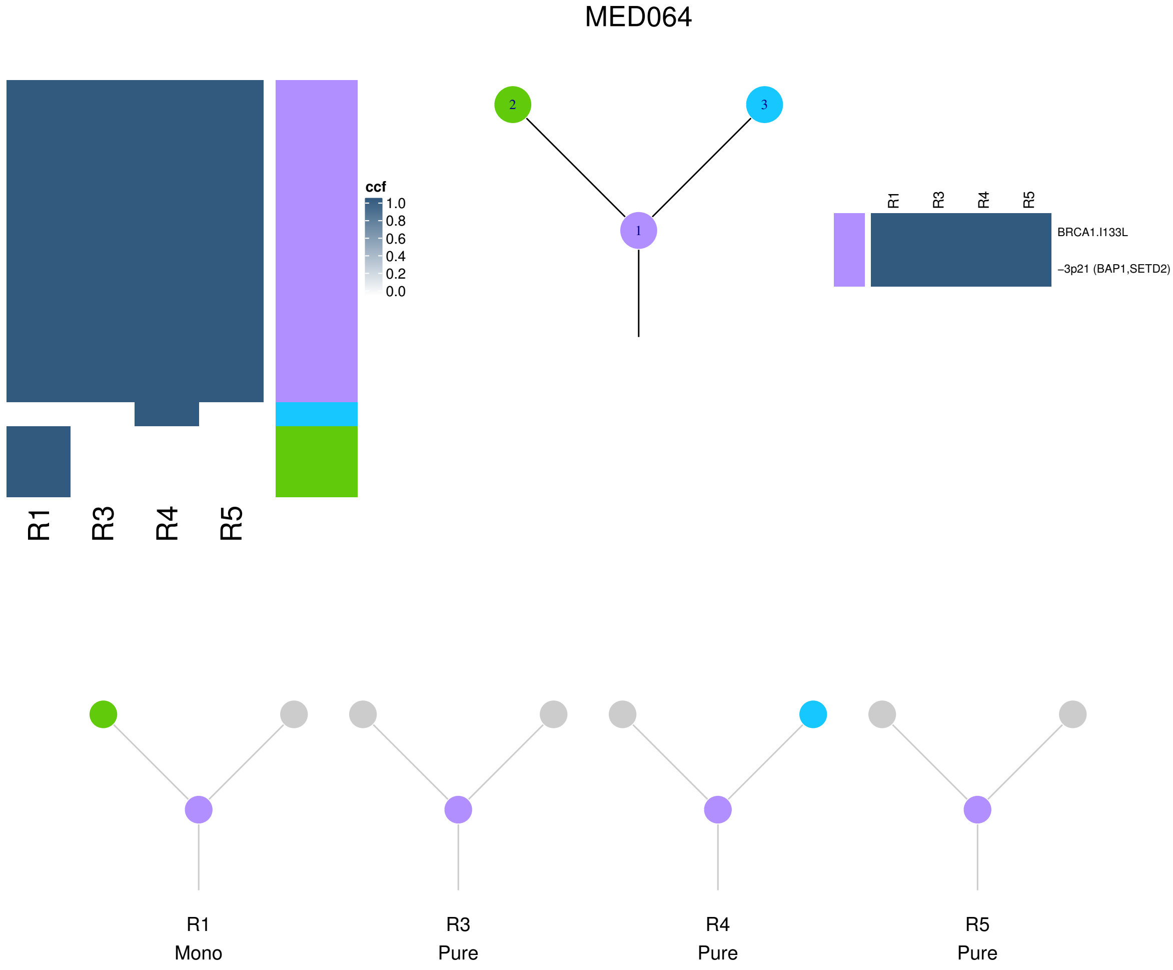

MED064
ccf
1.0
0.8
0.6
0.4
0.2
0.0
R1
R3
R4
R5
2
3
1
BRCA1.I133L
−3p21 (BAP1,SETD2)
R1
Mono
R3
Pure
R4
Pure
R5
Pure

O

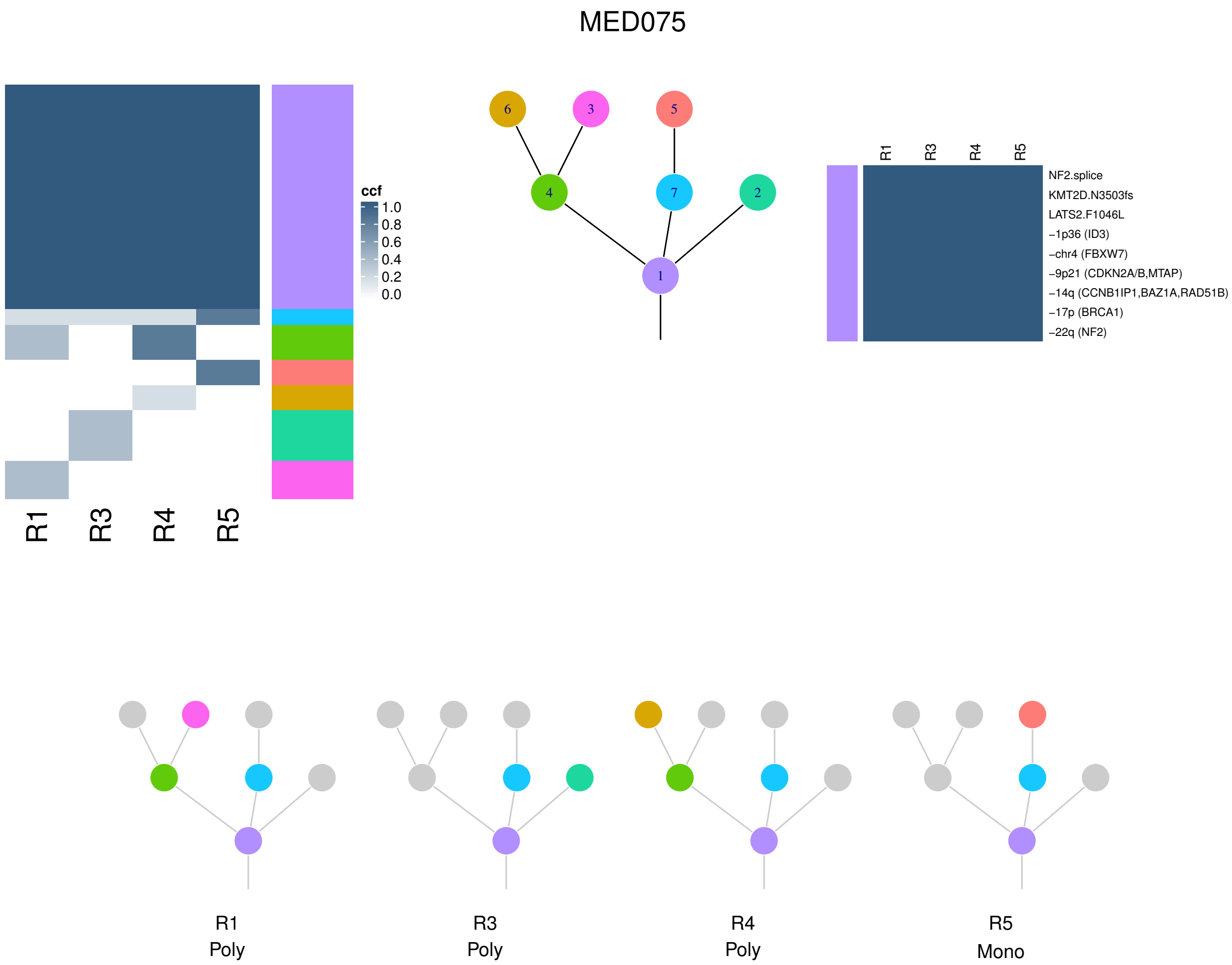
MED075
ccf
1.0
0.8
0.6
0.4
0.2
0.0
R1
R3
R4
R5
6
3
5
4
7
2
1
R1
R3
R4
R5
NF2.splice
KMT2D.N3503fs
LATS2.F1046L
−1p36 (ID3)
−chr4 (FBXW7)
−9p21 (CDKN2A/B,MTAP)
−14q (CCNB1IP1,BAZ1A,RAD51B)
−17p (BRCA1)
−22q (NF2)
R1
Poly
R3
Poly
R4
Poly
R5
Mono

P

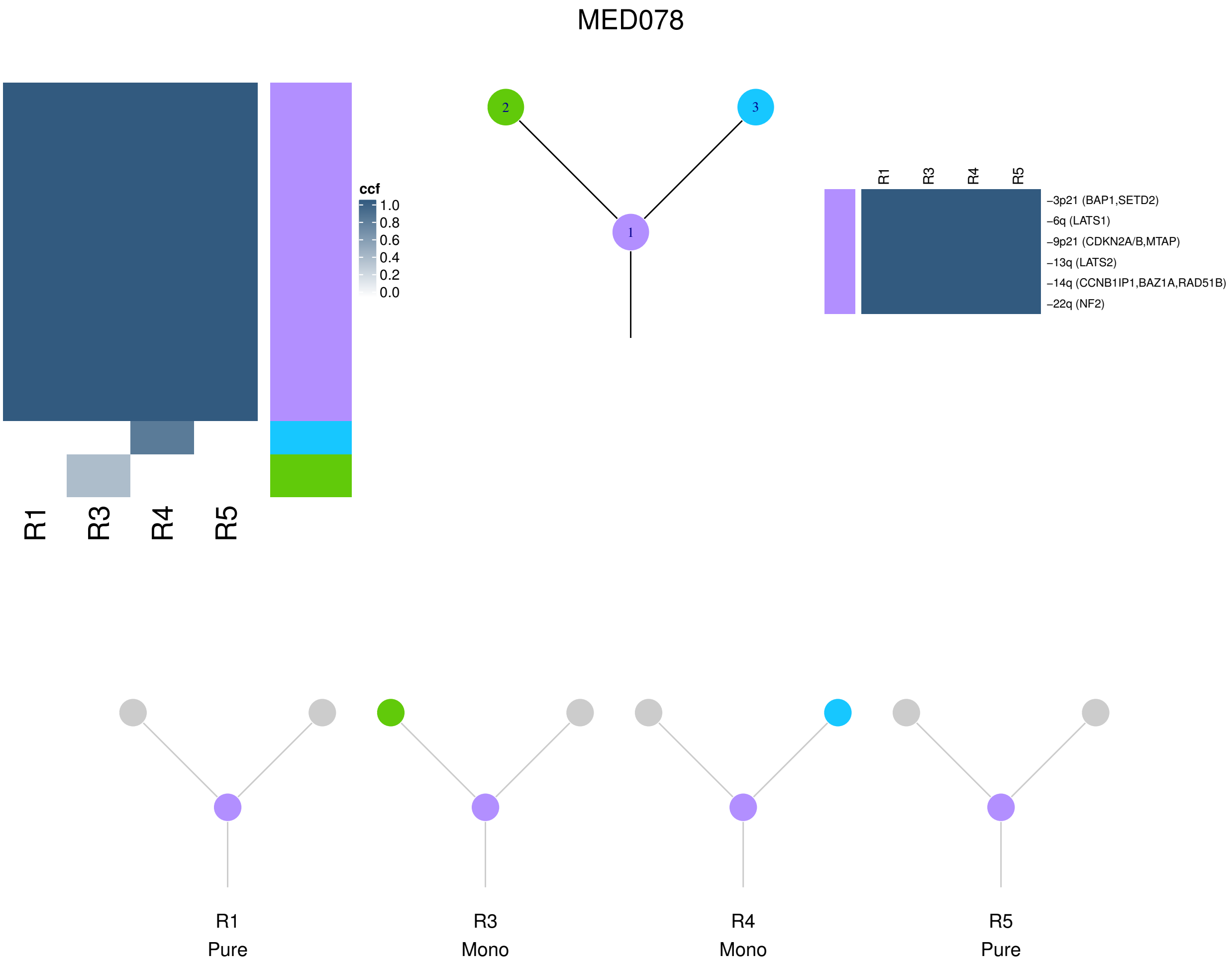
MED078
ccf
1.0
0.8
0.6
0.4
0.2
0.0
R1
R3
R4
R5
2
3
1
R1
R3
R4
R5
−3p21 (BAP1,SETD2)
−6q (LATS1)
−9p21 (CDKN2A/B,MTAP)
−13q (LATS2)
−14q (CCNB1IP1,BAZ1A,RAD51B)
−22q (NF2)
R1
Pure
R3
Mono
R4
Mono
R5
Pure

Q

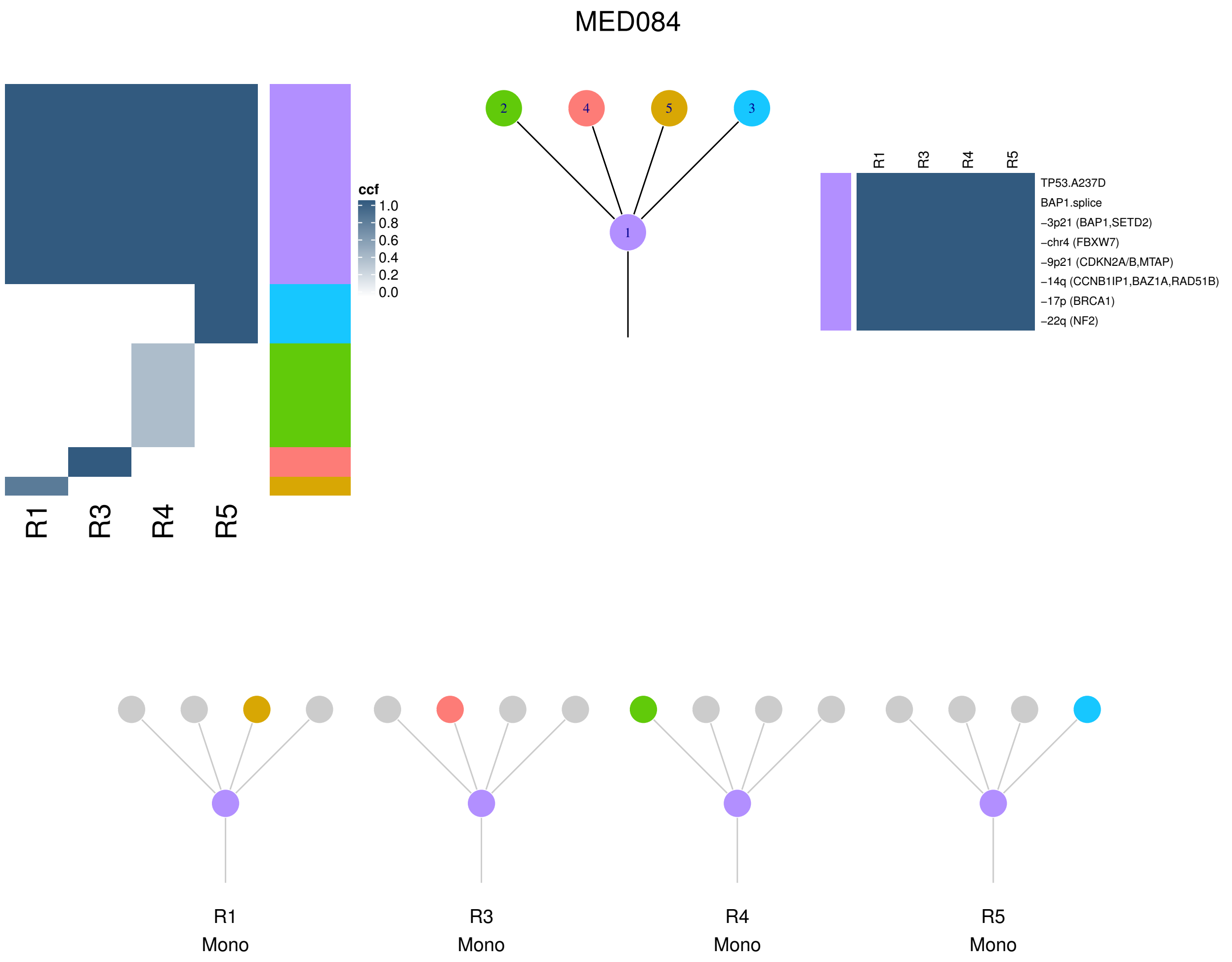
MED084
ccf
1.0
0.8
0.6
0.4
0.2
0.0
R1
R3
R4
R5
1
2
3
4
5
TP53.A237D
BAP1.splice
−3p21 (BAP1,SETD2)
−chr4 (FBXW7)
−9p21 (CDKN2A/B,MTAP)
−14q (CCNB1IP1,BAZ1A,RAD51B)
−17p (BRCA1)
−22q (NF2)
R1
Mono
R3
Mono
R4
Mono
R5
Mono

R

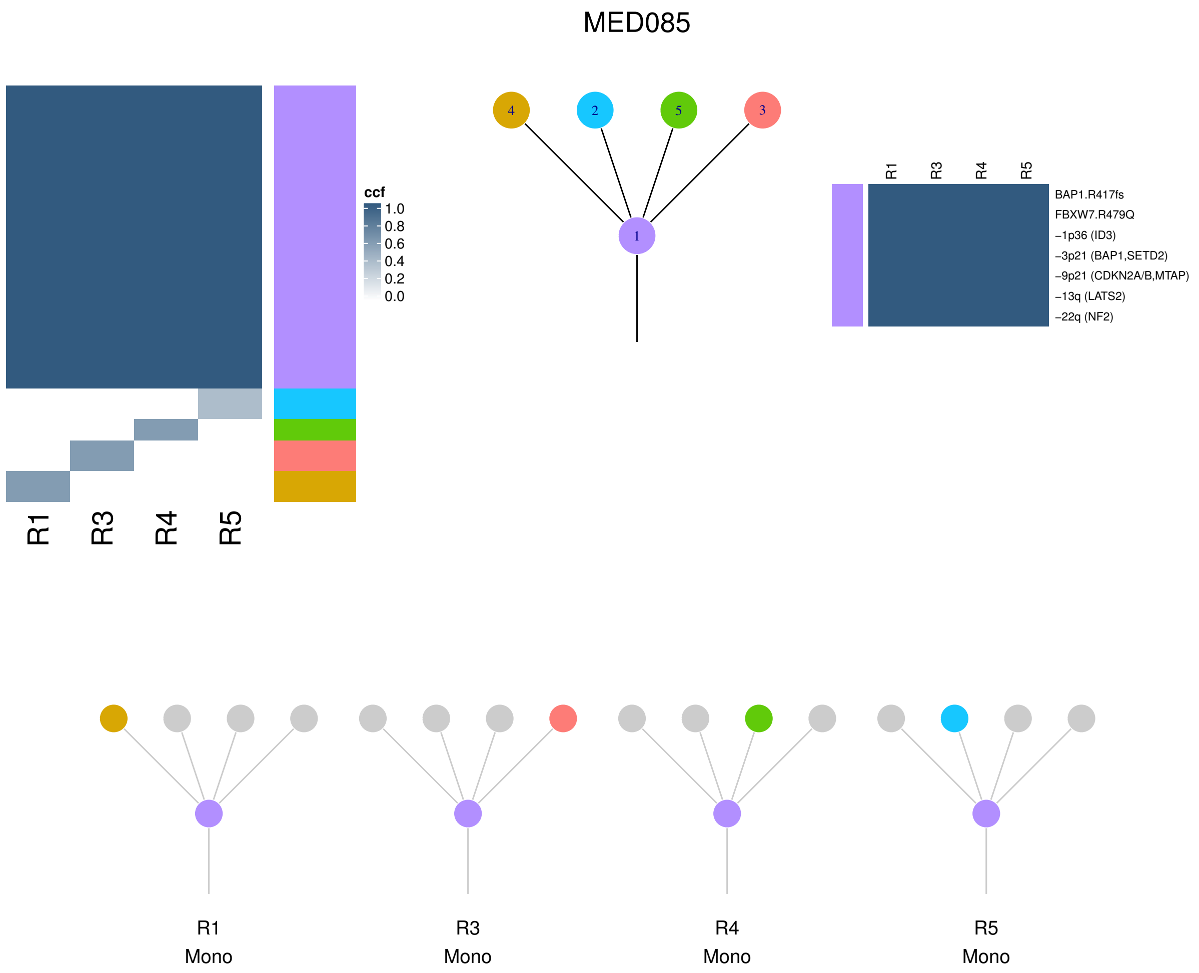
MED085
ccf
1.0
0.8
0.6
0.4
0.2
0.0
R1
R3
R4
R5
1
2
3
4
5
BAP1.R417fs
FBXW7.R479Q
−1p36 (ID3)
−3p21 (BAP1,SETD2)
−9p21 (CDKN2A/B,MTAP)
−13q (LATS2)
−22q (NF2)
R1
Mono
R3
Mono
R4
Mono
R5
Mono

S

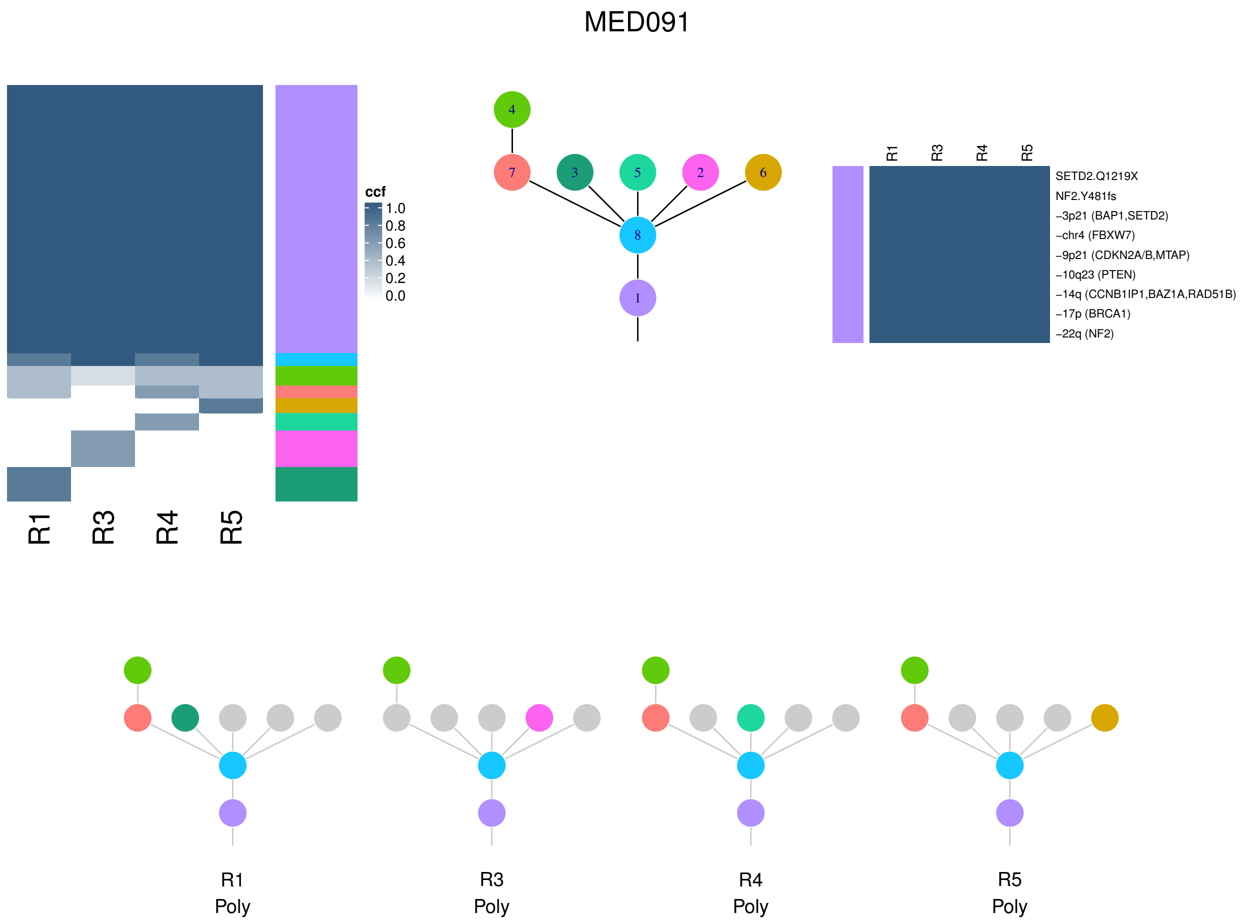
MED091
ccf
1.0
0.8
0.6
0.4
0.2
0.0
R1
R3
R4
R5
SETD2.Q1219X
NF2.Y481fs
−3p21 (BAP1,SETD2)
−chr4 (FBXW7)
−9p21 (CDKN2A/B,MTAP)
−10q23 (PTEN)
−14q (CCNB1IP1,BAZ1A,RAD51B)
−17p (BRCA1)
−22q (NF2)
R1
Poly
R3
Poly
R4
Poly
R5
Poly

T

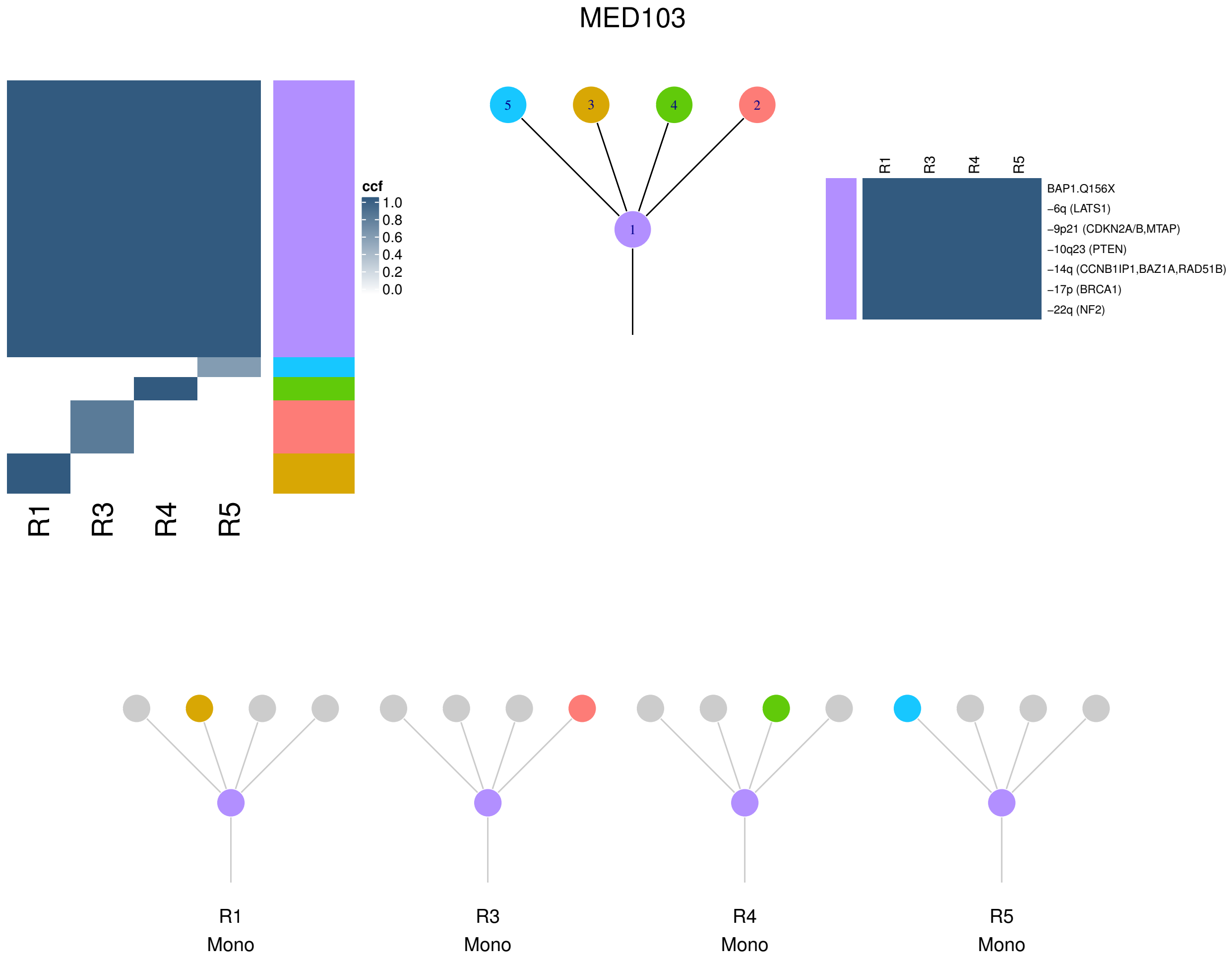
MED103
ccf
1.0
0.8
0.6
0.4
0.2
0.0
R1
R3
R4
R5
BAP1.Q156X
−6q (LATS1)
−9p21 (CDKN2A/B,MTAP)
−10q23 (PTEN)
−14q (CCNB1IP1,BAZ1A,RAD51B)
−17p (BRCA1)
−22q (NF2)
R1
Mono
R3
Mono
R4
Mono
R5
Mono

U

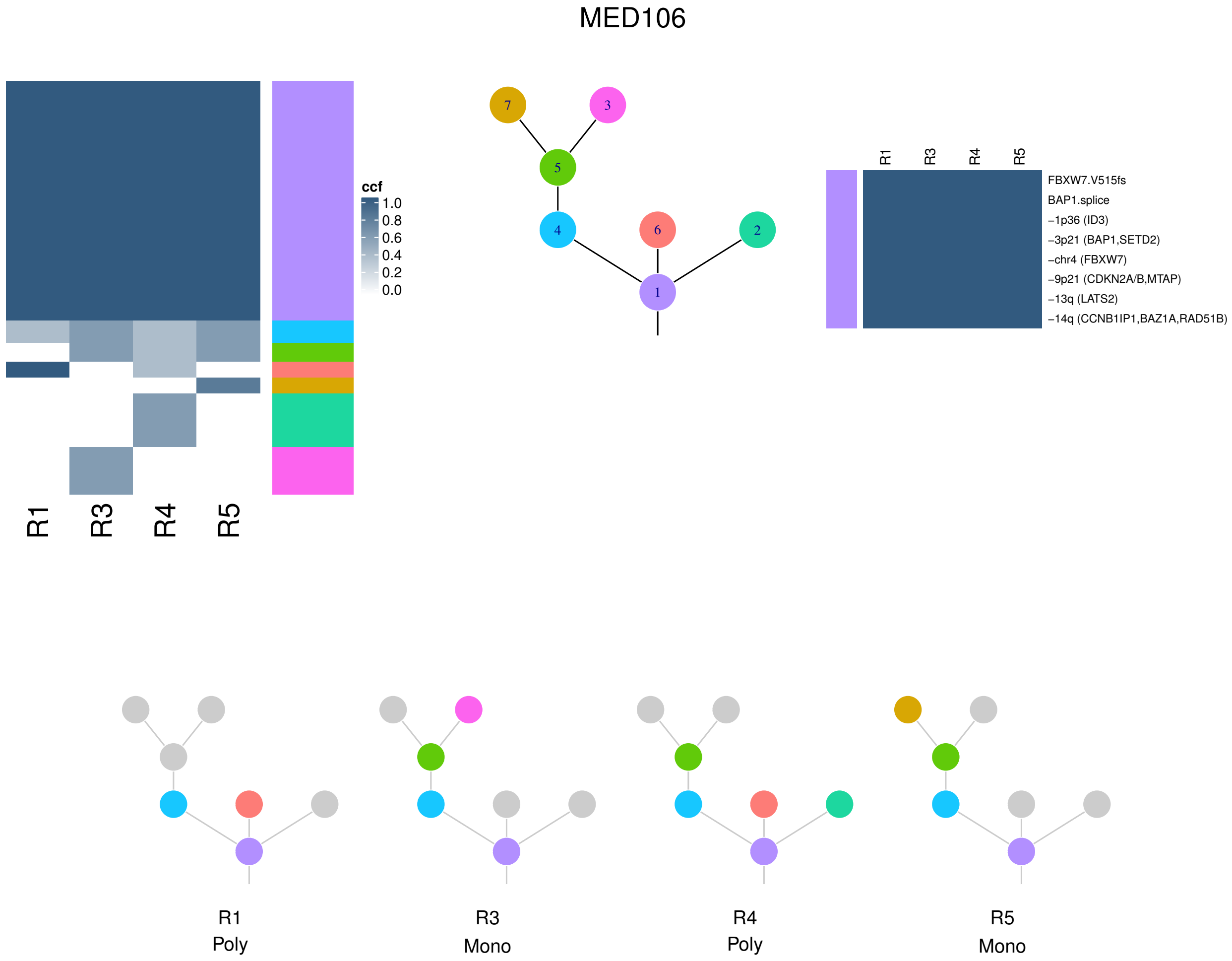
MED106
ccf
1.0
0.8
0.6
0.4
0.2
0.0
R1
R3
R4
R5
7
3
5
4
6
2
1
FBXW7.V515fs
BAP1.splice
−1p36 (ID3)
−3p21 (BAP1,SETD2)
−chr4 (FBXW7)
−9p21 (CDKN2A/B,MTAP)
−13q (LATS2)
−14q (CCNB1IP1,BAZ1A,RAD51B)
R1
Poly
R3
Mono
R4
Poly
R5
Mono

V

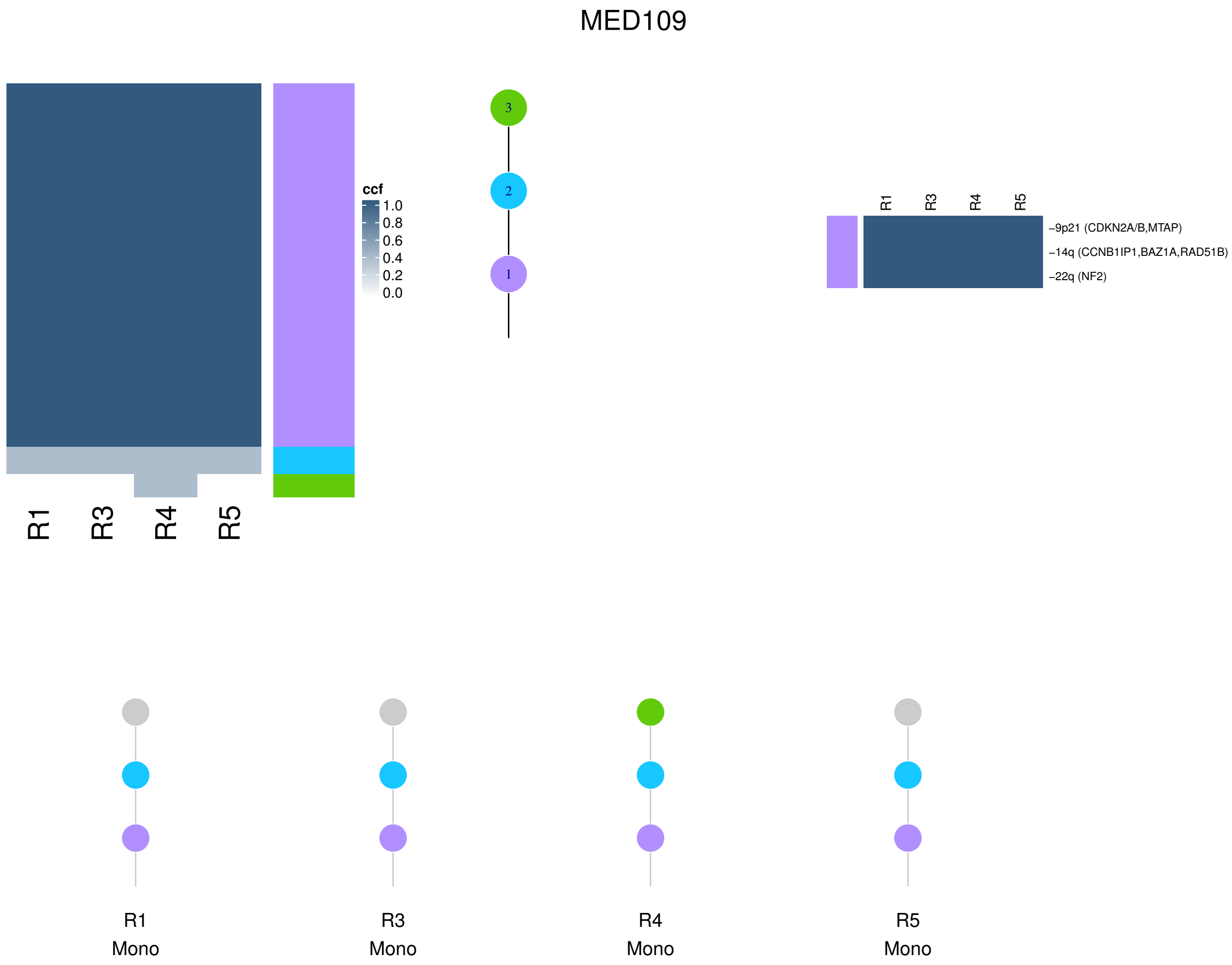
MED109
ccf
1.0
0.8
0.6
0.4
0.2
0.0
R1
R3
R4
R5
3
2
1
−9p21 (CDKN2A/B,MTAP)
−14q (CCNB1IP1,BAZ1A,RAD51B)
−22q (NF2)
R1
Mono
R3
Mono
R4
Mono
R5
Mono

Supplementary Figure 3. Regional clonal deconvolution

Phylogenetic trees for the MEDUSA22 cohort. For each tumour, top-left shows cancer cell fractions (CCF) as a heatmap for all clustered mutations. CCF value for each mutation represents the mean of the mutation cluster CCF values, with darker colour indicating higher CCF value. Middle top panel shows the complete phylogenetic tree as constructed based on the mutation clusters. Driver mutations and SCNAs are shown on top-right panel. Below are phylogenetic trees drawn for each region, with clusters not found in a given region shown in grey, and phylogentic categories are shown with the region names.

Supplementary Figure 4

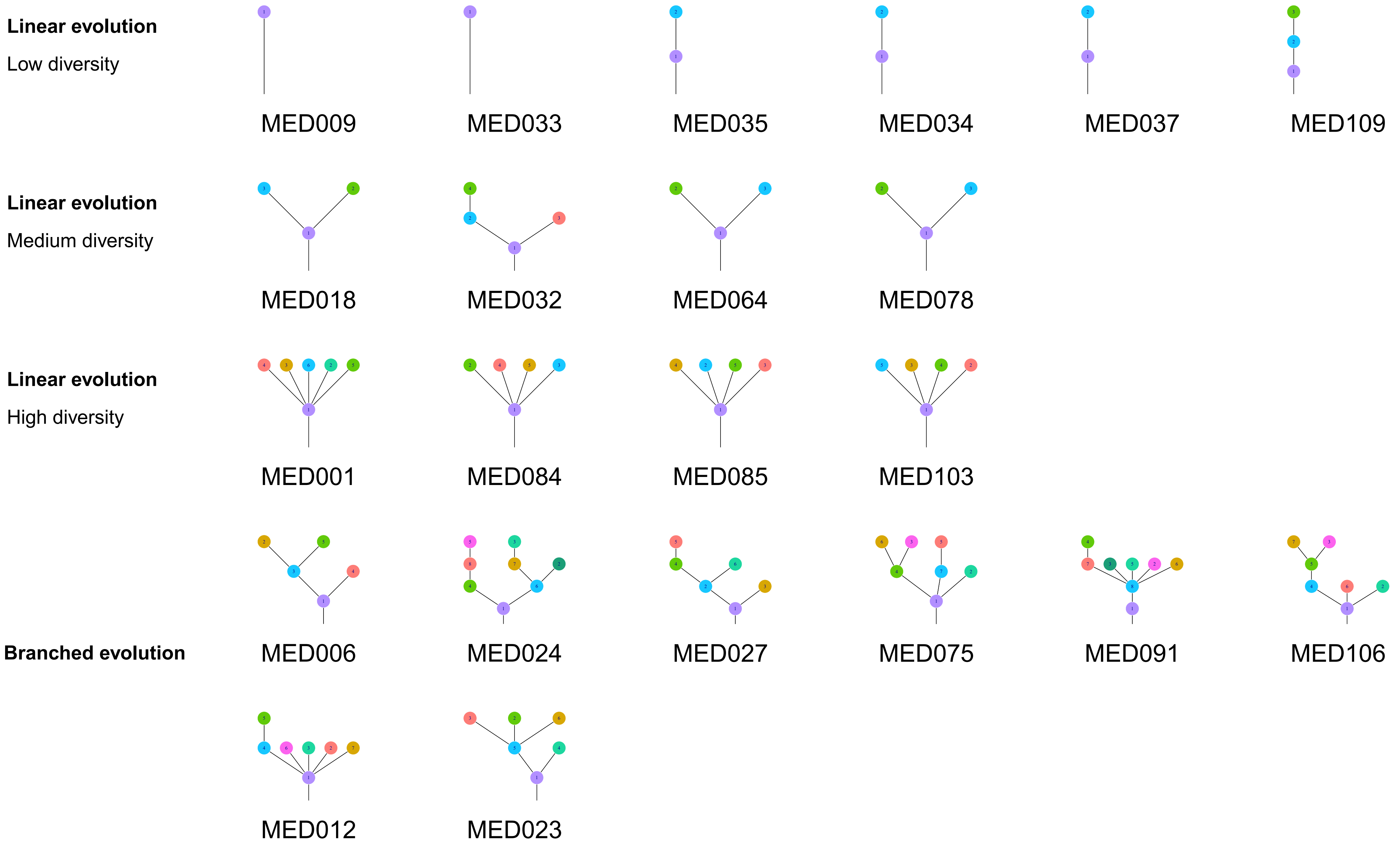

Supplementary Figure 4. Evolutionary diversity across the MEDUSA22 cohort

Linear and branched evolutionary models in MEDUSA22 determined by clonal deconvolution. Evolutionary models were into two broad categories, linear or branched, based on the presence or absence of subclonal branching in the phylogenetic trees. Linear model was separated into 3 categories further subcategories: low diversity corresponding to no branching, medium diversity corresponding to no more than two branches, and high diversity corresponding to more than two branches. In contrast, branched evolution was divided into non-truncal subclonal (where according to their phylogeny diversity. Branched model exhibited the highest level of diversity defined by the presence of subclonal diversification involving two or more branches.

Supplementary Figure 5

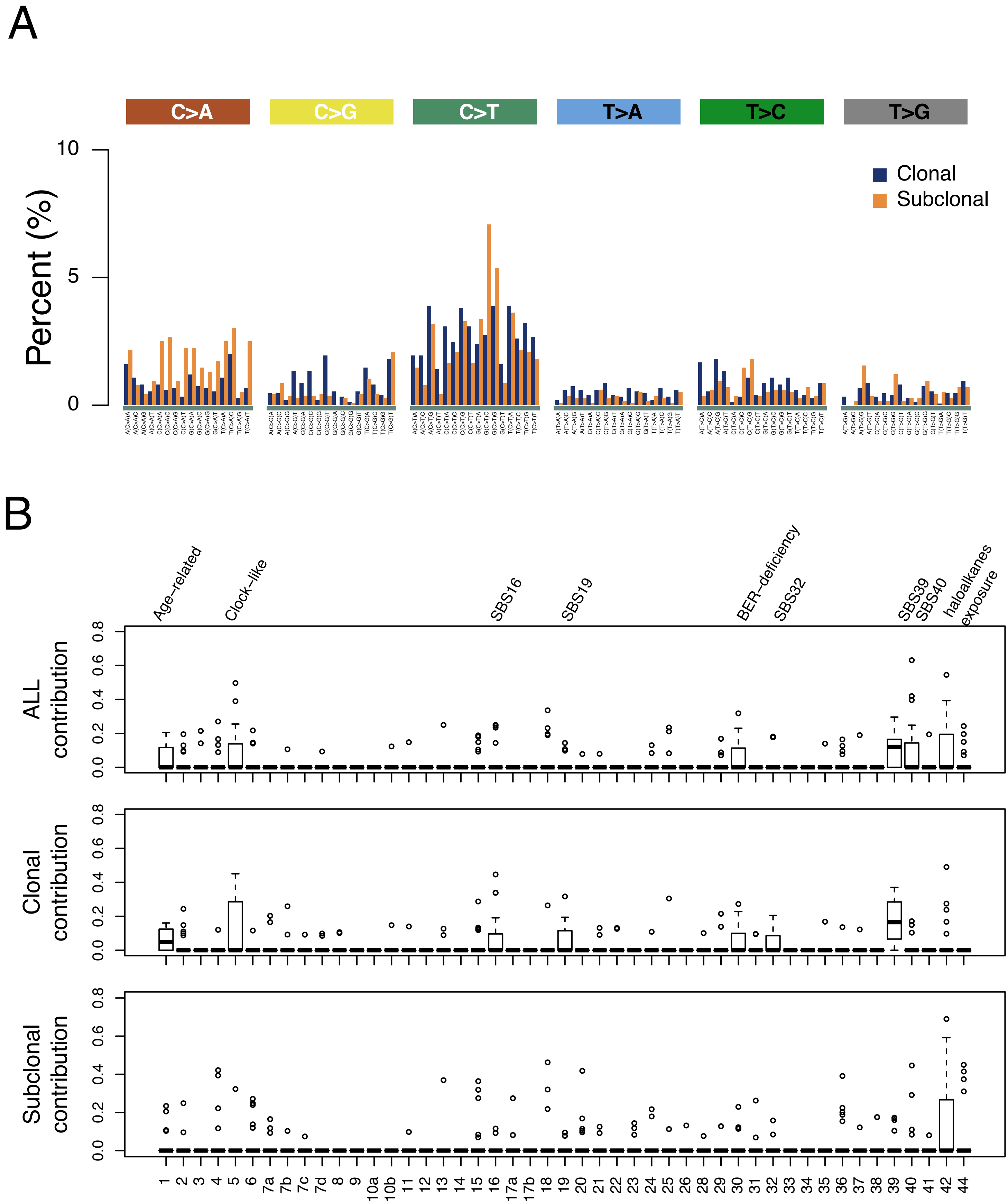

Supplementary Figure 5. Mutation signatures in the MEDUSA22 cohort

Mutational signatures identified based on evolutionary timing. (A) Proportion of SNVs occurring in specific nucleotide motif contexts for each category of single nucleotide substitution COSMIC V3 signatures were deconstructed by deconstructSigs and plotted as boxplots (B) for clonal and subclonal SNVs.

Supplementary Figure 6

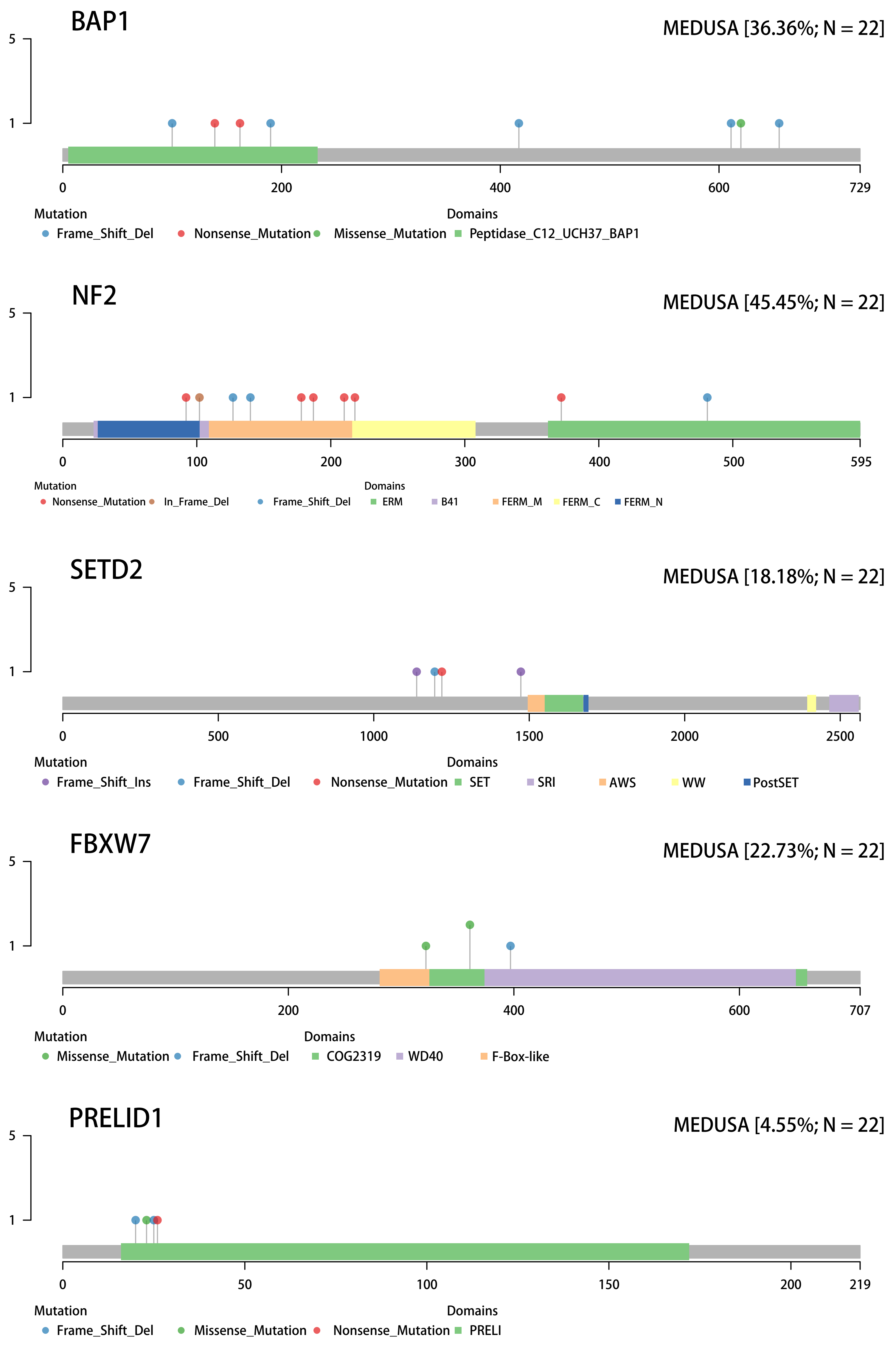

Supplementary Figure 6. Clonal driver mutations

Lollipop plots of mutations involving positively selected genes: *BAP1*, *NF2*, *SETD2*, *FBXW7* and *PRELID1*. The related frequency of mutations involving these genes is shown with the denominator (22 patients were studied).

Supplementary Figure 7

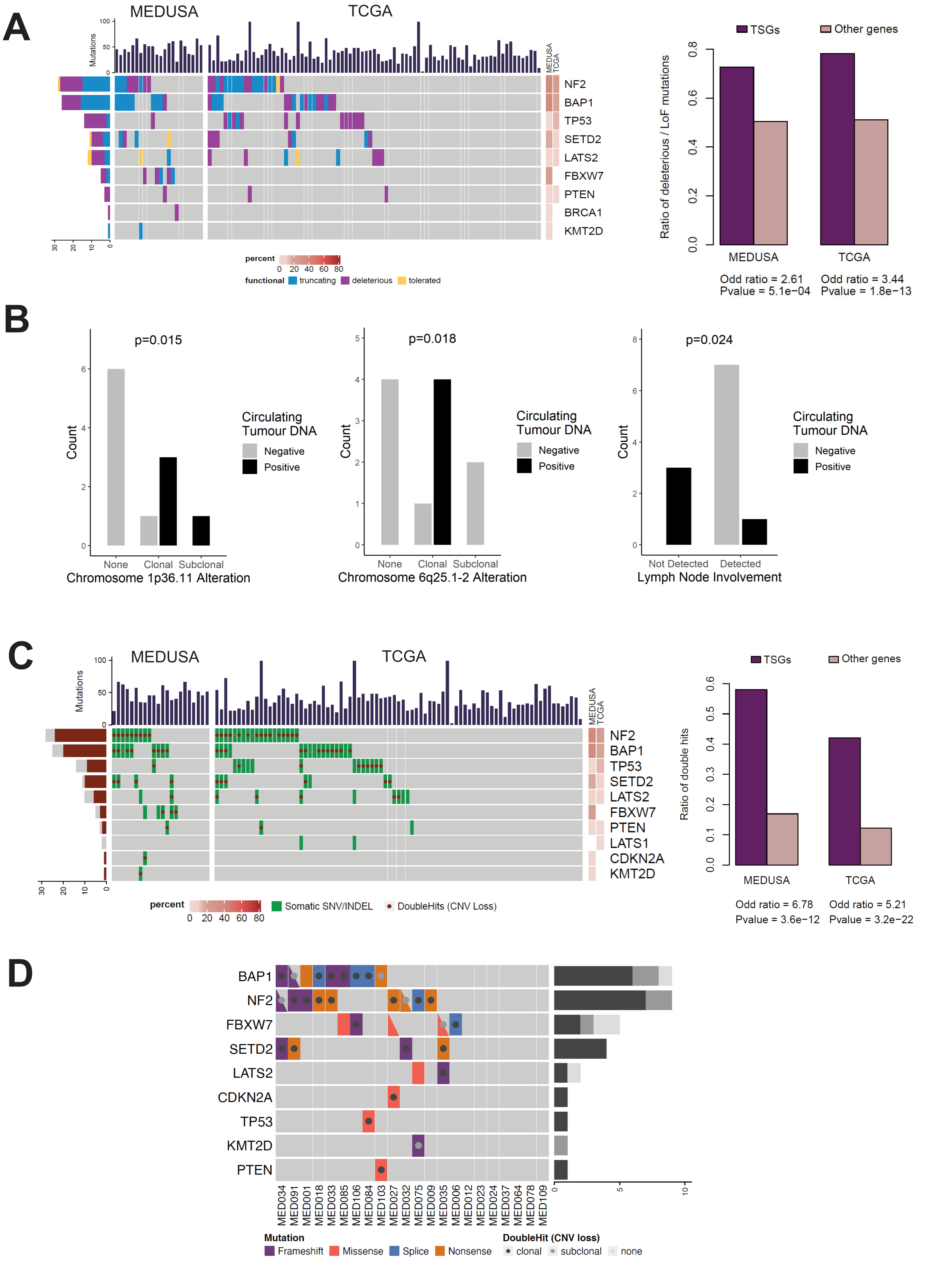

Supplementary Figure 7. Deleterious mutations and detection in circulating DNA

A. Heatmap showing deleterious or tolerated mutations in the MEDUSA22 cohort versus TCGA cohorts. Right panel. Histogram showing the ratio of deleterious to loss of function mutations in the MEDUSA22 and TCGA cohorts showing an excess of deleterious mutations affecting tumour suppressor genes (TSGs). Mutations show a similar rank order between the MEDUSA22 and TCGA cohorts.

B. Column charts showing the relative number of patients with positive circulating tumour DNA harbouring 1p36.11, 6q25.1-2 alterations as detected by whole exome sequencing or lymph node involvement. The column charts show an imbalance with ctDNA detected (black) in mutated compared to non-mutated patients. However, lymph node involvement is associated with increased ctDNA negative plasma. n=11 patients.

C. Heatmap summarising double hit events in the MEDUSA 22 and TCGA cohorts involving secondary copy number loss leading to bi-allelic inactivation. Right panel. Histograms showing the relative ratio of double hits involving TSGs versus other genes. Double-hit events show a similar rank order between the MEDUSA22 and TCGA cohorts.

D. Heatmap summarising clonal versus subclonal relationships for double hits involving frequently altered cancer drivers.

Supplementary Figure 8

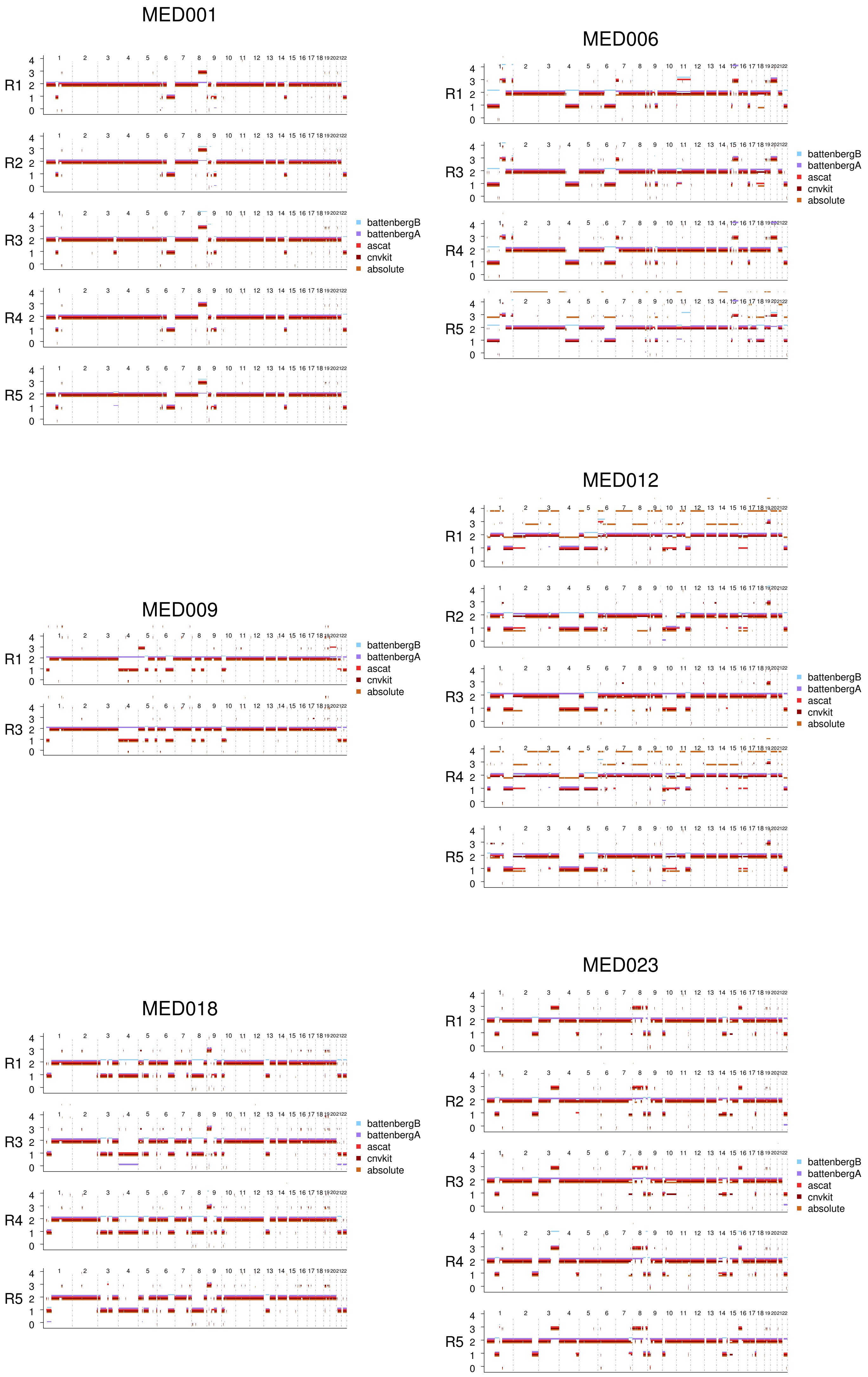

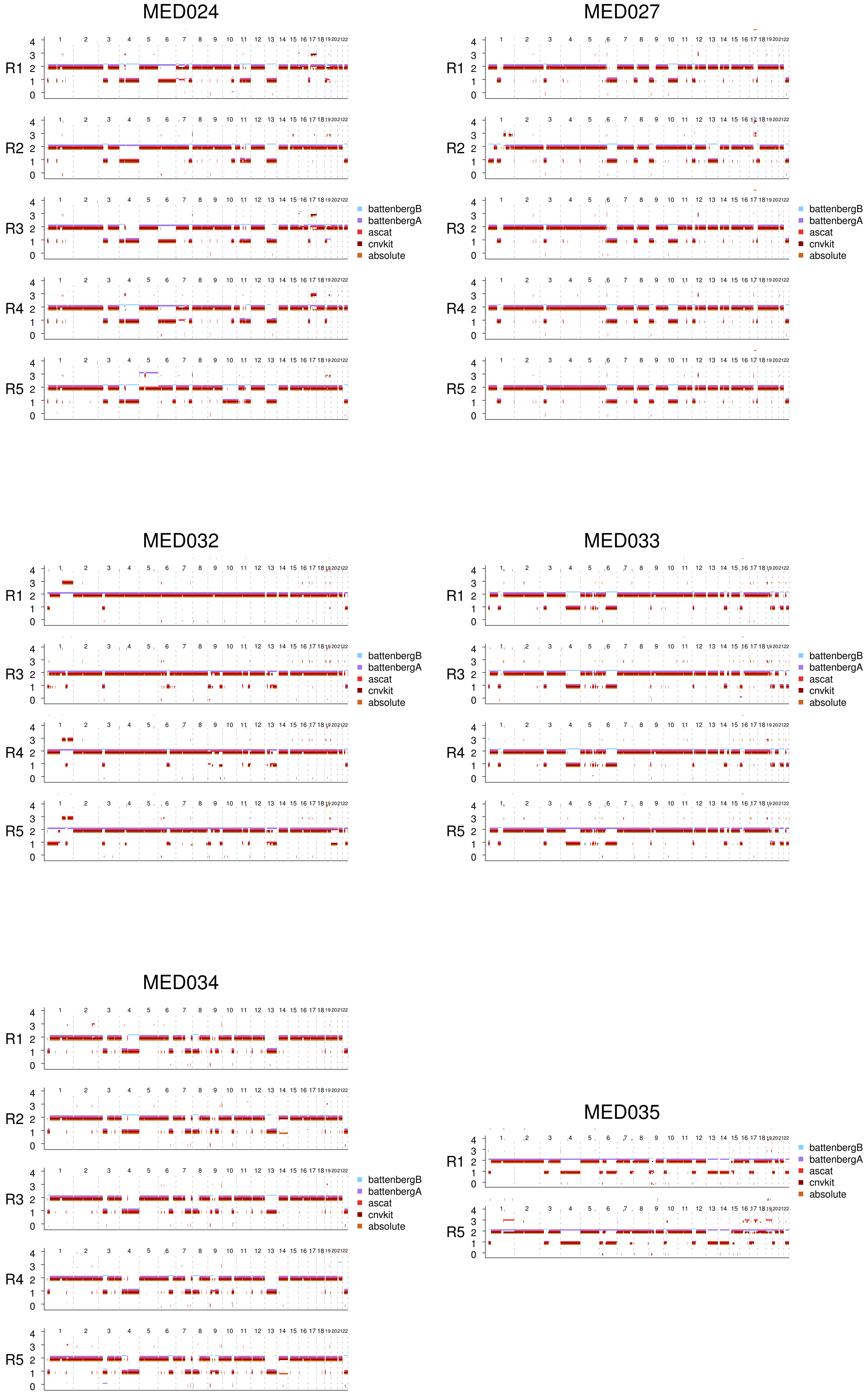
MED024
MED027
MED032
MED033
MED034
MED035
R1
R2
R3
R4
R5
battenbergB
battenbergA
ascat
cnvkit
absolute

MED037

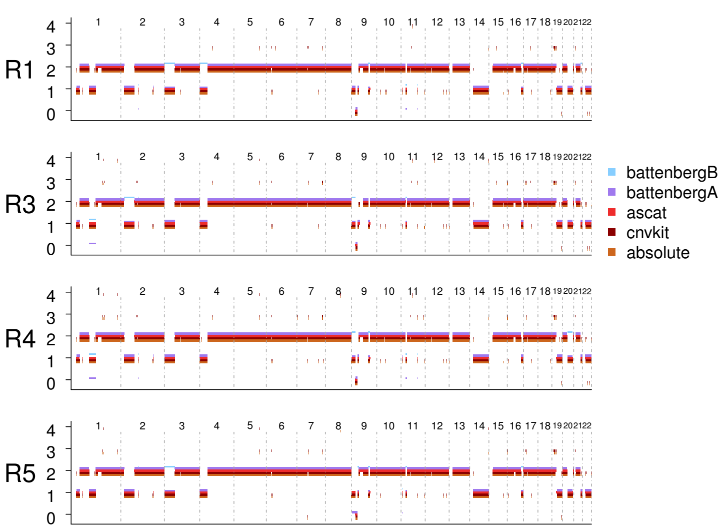

MED064

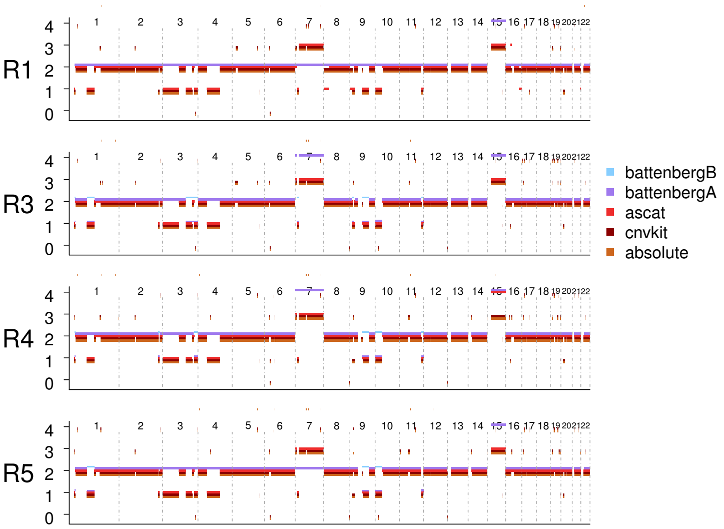

MED075

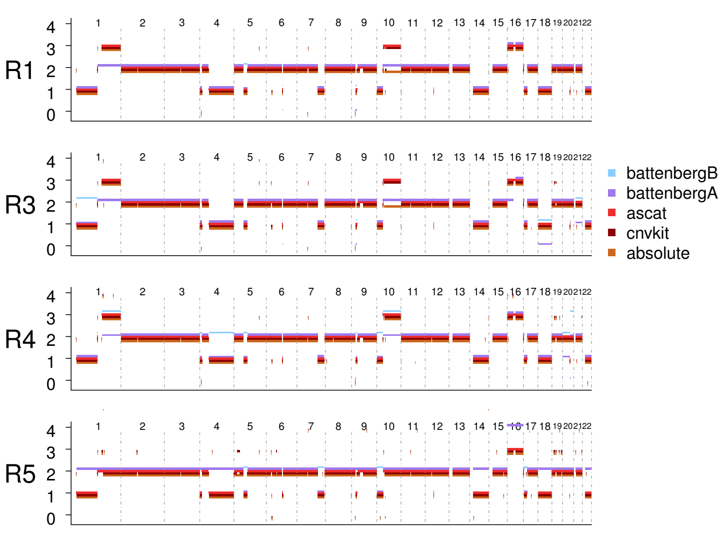

MED078

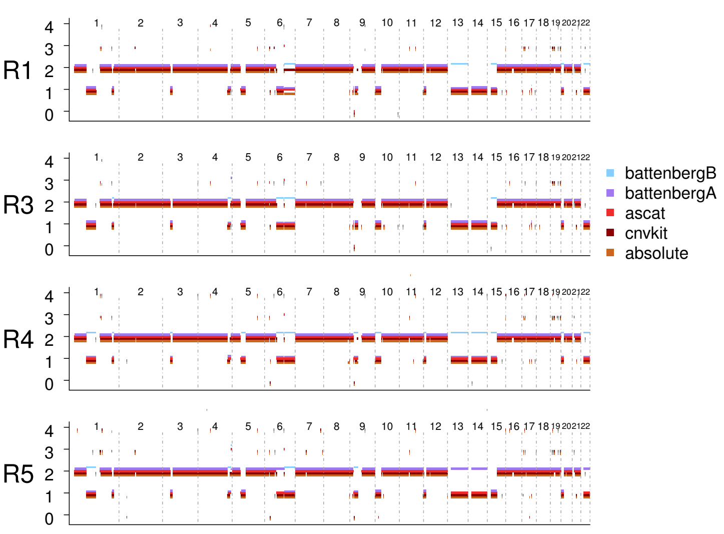

MED084

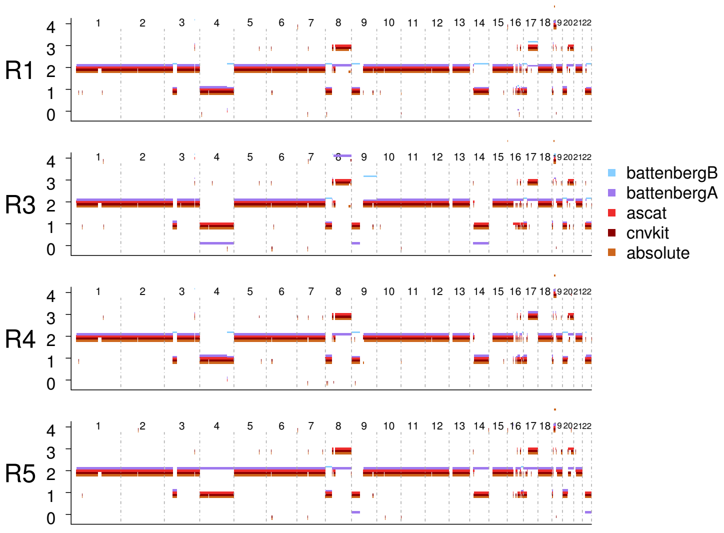

MED085

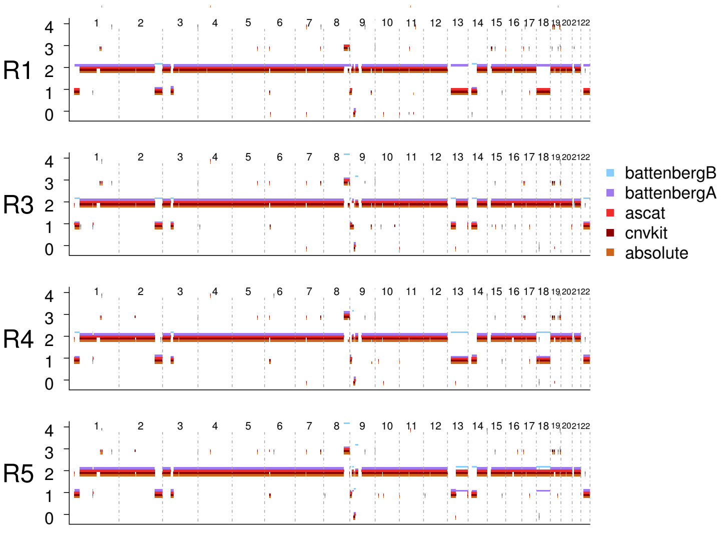

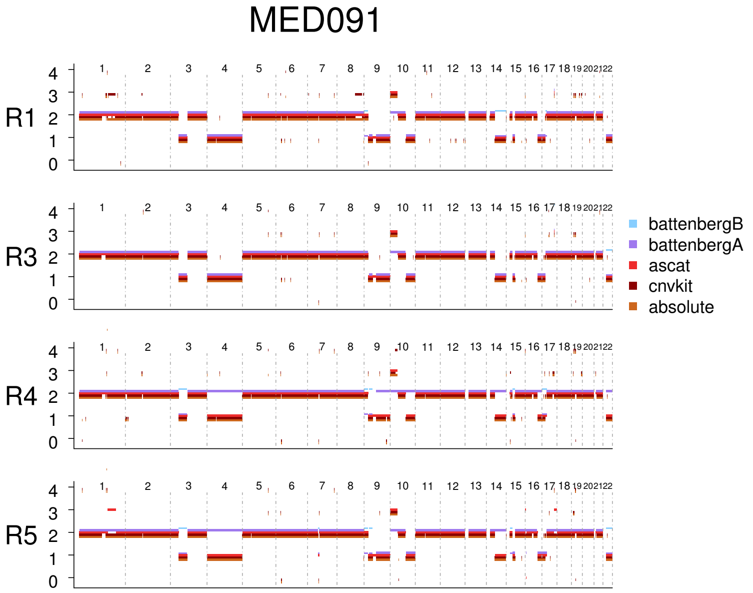
MED091
R1
R3
R4
R5
1 2 3 4 5 6 7 8 9 10 11 12 13 14 15 16 17 18 19 20 21 22
0 1 2 3 4
battenbergB
battenbergA
ascat
cnvkit
absolute

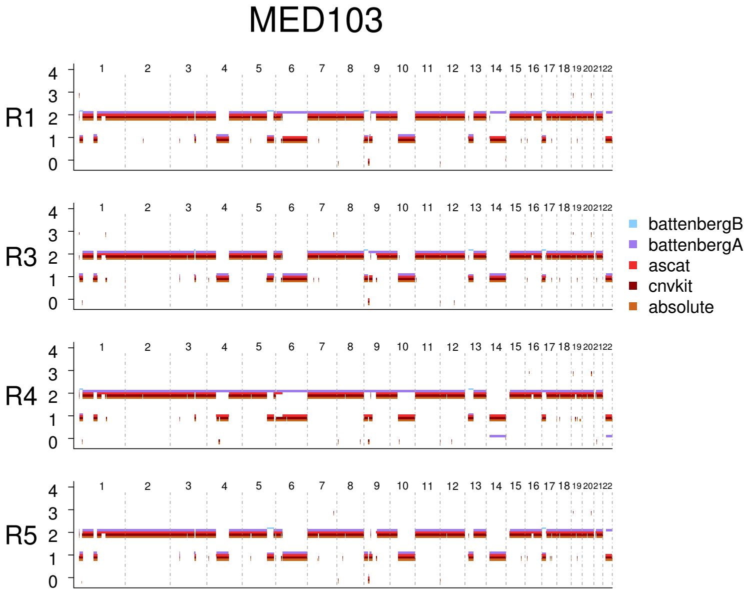
MED103
R1
R3
R4
R5
1 2 3 4 5 6 7 8 9 10 11 12 13 14 15 16 17 18 19 20 21 22
0 1 2 3 4
battenbergB
battenbergA
ascat
cnvkit
absolute

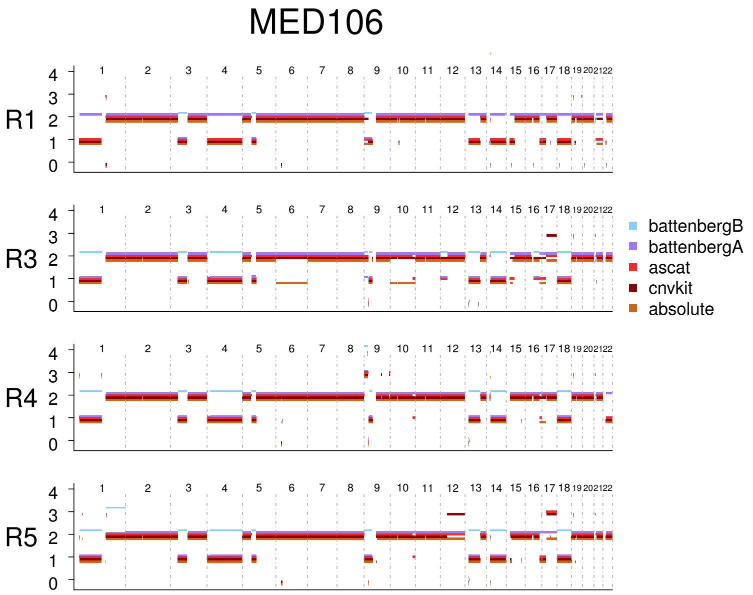
MED106
R1
R3
R4
R5
1 2 3 4 5 6 7 8 9 10 11 12 13 14 15 16 17 18 19 20 21 22
0 1 2 3 4
battenbergB
battenbergA
ascat
cnvkit
absolute

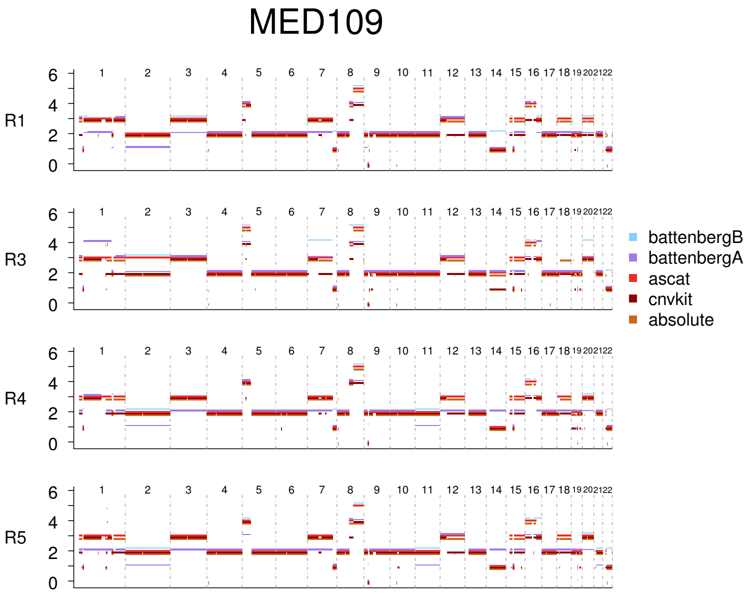
MED109
R1
R3
R4
R5
1 2 3 4 5 6 7 8 9 10 11 12 13 14 15 16 17 18 19 20 21 22
0 2 4 6
battenbergB
battenbergA
ascat
cnvkit
absolute

Supplementary Figure 8. Copy number alterations per region

Comparison of multiple SCNA algorithms. For each tumour-normal pair, segmented log2ratio data was generated by CNVkit. Then purity, ploidy and absolute copy numbers were estimated by ABSOLUTE based on somatic mutations and the segmented data, and CNVkit utilized the estimated purity and ploidy to call copy number. Besides, ASCAT and Battenberg was applied to detect allelic specific copy number variations. All the samples show high concordance of copy number profiles among these methods except MED006-R5, MED012-R1 and MED012-R4, which has no best solutions in ABSOLUTE. The following factors explain the number of regions presented in Supplementary Figure 8. This figure presents evidence of concordance between 5 different somatic copy number alteration (SCNA) calling algorithms across a subset of patients. The different sample numbers are explained in part due to the fact that some patients had between 4 and 5 regions sequenced (table S2). MED009, MED035 shows only two regions as these regions met the stringent quality control for reporting in the study.

Supplementary Figure 9

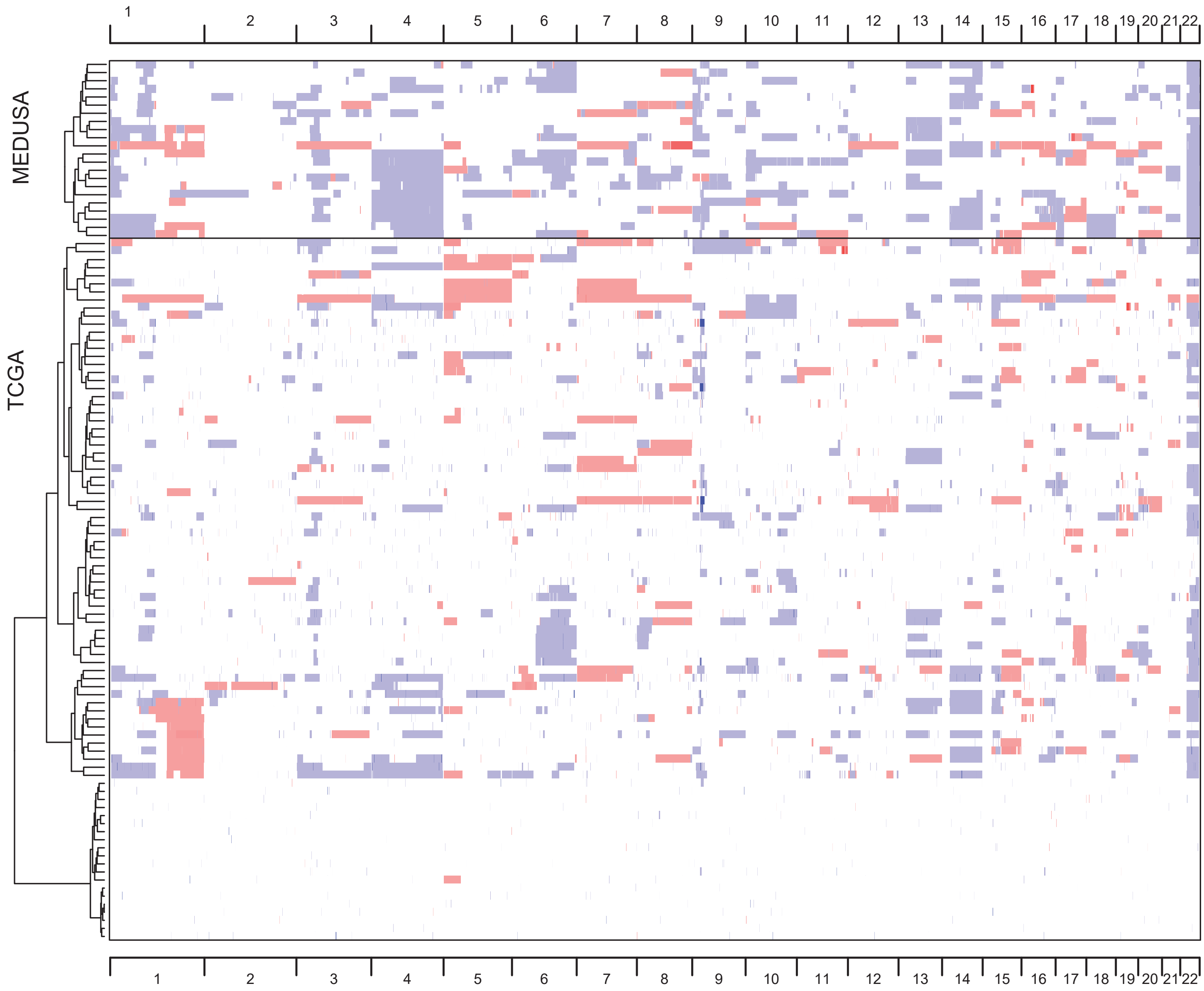

Supplementary Figure 9. Copy number landscape of MPM in MEDUSA22 versus TCGA

Comparative distribution of somatic copy number alterations (SNCA) in the MEDUSA22 versus the TCGA[7] cohorts. The x-axis represents the whole exome by chromosome number (1 through 22). The presence of a loss is denoted in blue and a gain in red. SCNAs show a similar distribution between the MEDUSA22 and TCGA cohorts.

Supplementary Figure 10

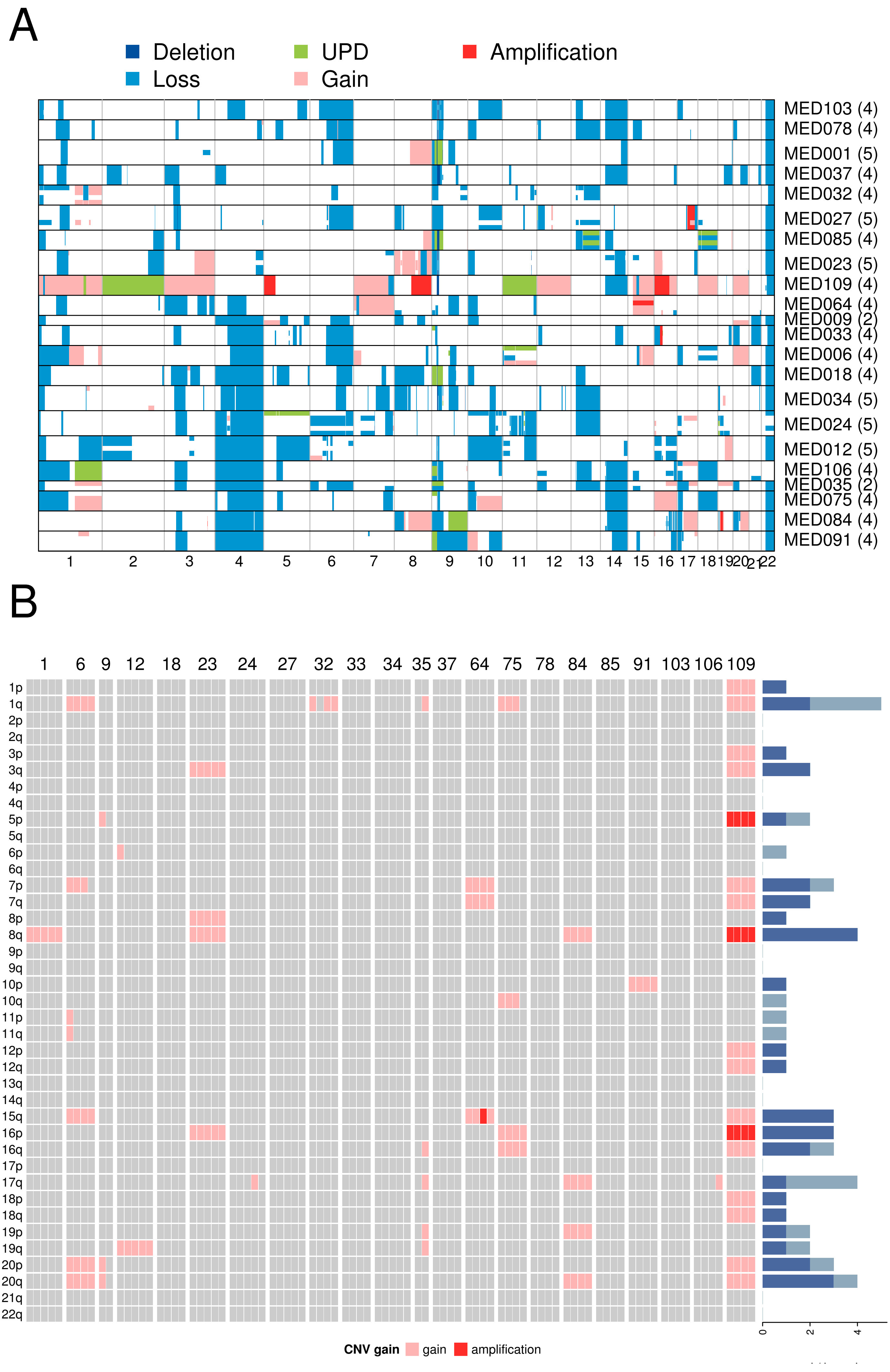

Supplementary Figure 10 Copy number gains

(A) Gains/amplifications, losses and uniparental disomy (UPD) detected in MEDUSA22. (B) Heatmap showing gains and amplification events ordered by chromosome (left column) and frequency histogram on the right. Although 4-5 samples per patient were sequenced, a very stringent filtering workflow was employed to ensure the quality of data generated from the whole exome sequencing. For one patient, MED035 only two of the 4 regions passed the quality control required for reporting and so this is shown in the figure.

Supplementary Figure 11

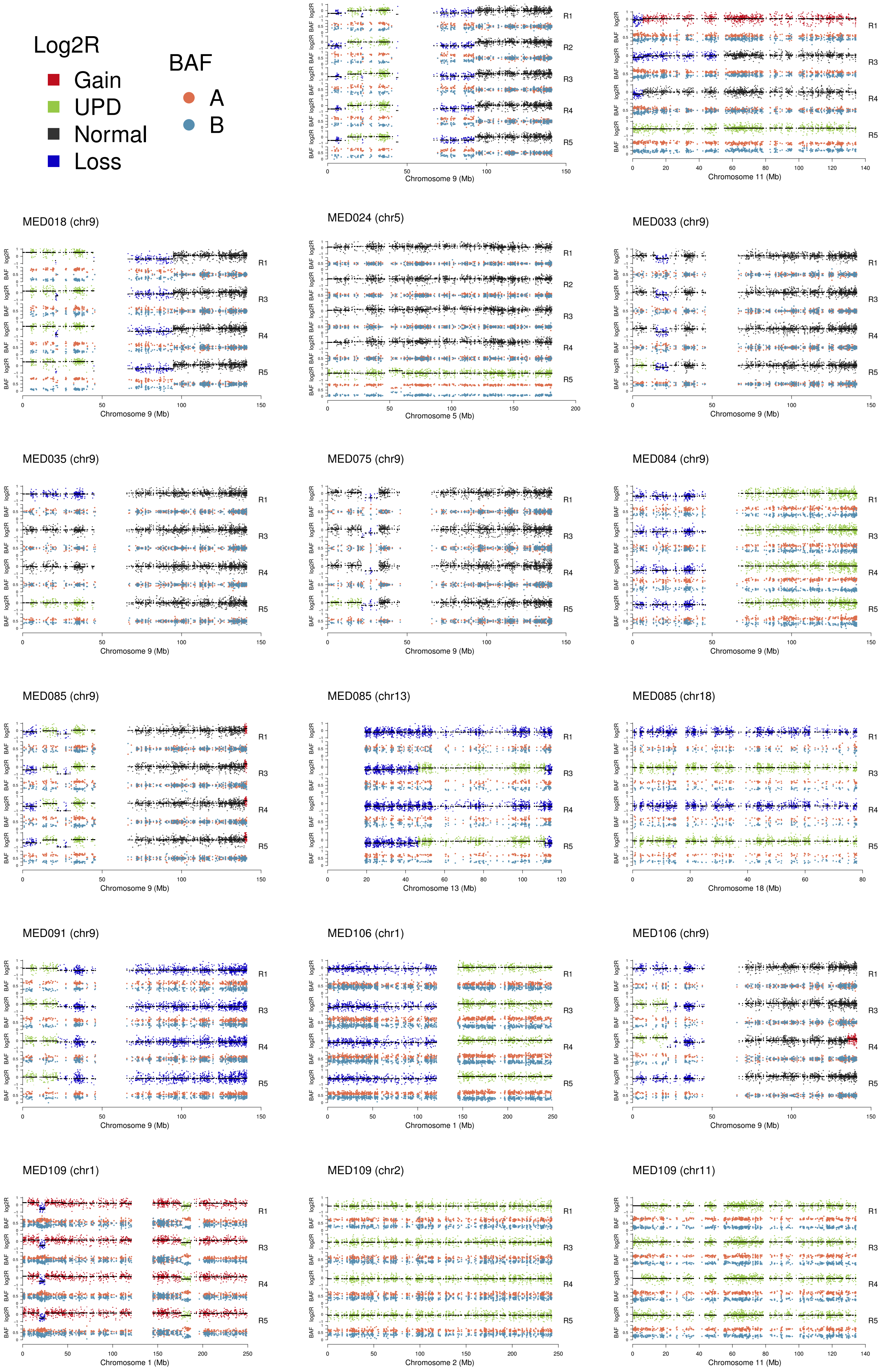

Supplementary Figure 11. UPD in the MEDUSA22 cohort

UPD (uniparental disomy, or CN LOH) detected in MEDUSA22. Following shows chromosome log2ratio and BAF profiles with UPD events. For each region, top panel shows probe-level log2ratio profile , in which red points indicate gain, green for UPD, grey for normal and blue for loss. In bottom panel, allele frequencies of heterozygous single nucleotide polymorphisms are plotted, in which red and blue points indicate the major or minor alleles.

Supplementary Figure 12

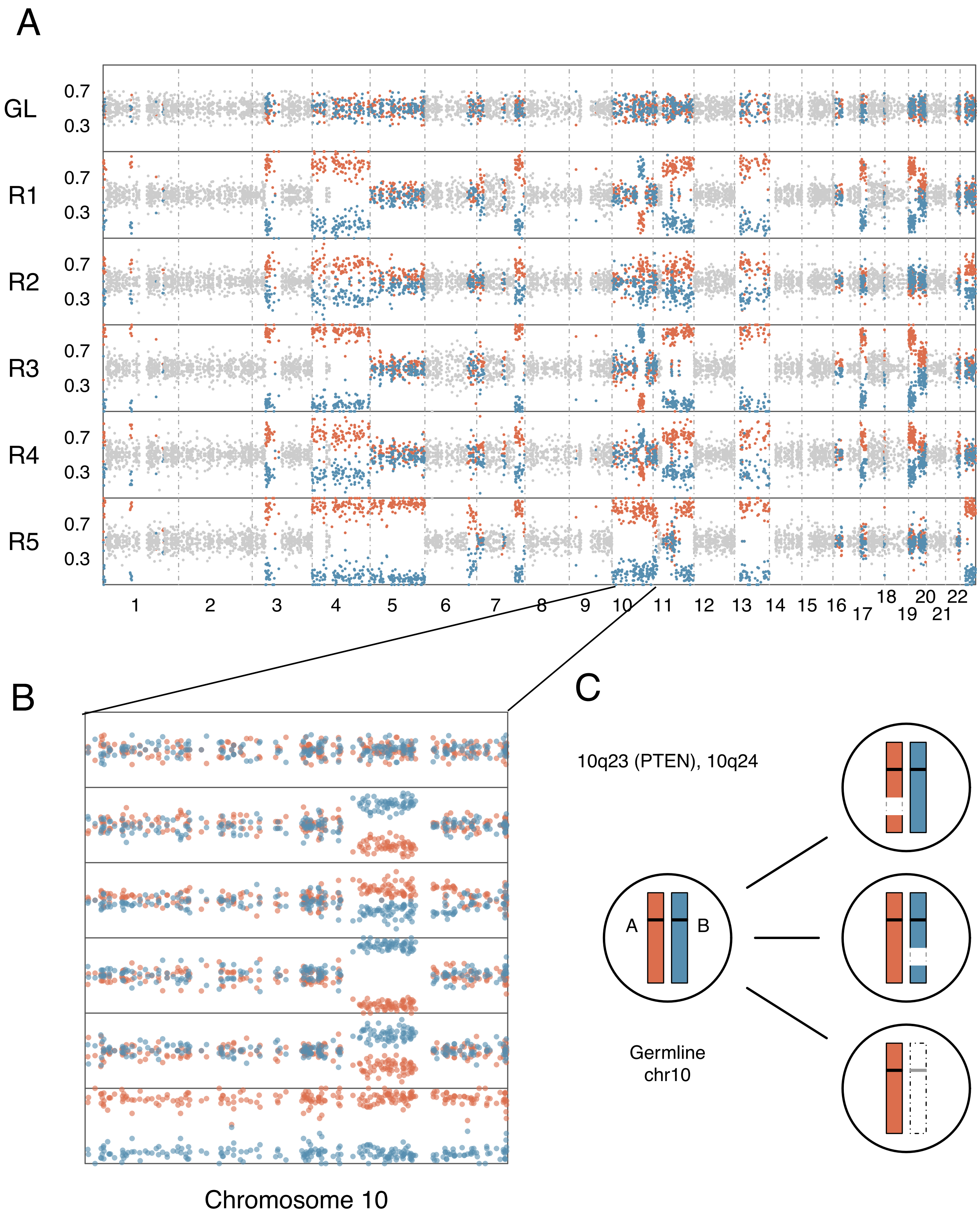

Supplementary Figure 12. Mirrored subclonal allelic imbalance in the MEDUSA22 cohort

Patient MED024 exhibits mirrored subclonal allelic imbalance events (MSAI). BAF profiles of germline (GL) and five tumour regions show high diversity of allelic specific copy number alterations across the genome, such as chr5, chr6, chr10, chr17, chr19 and chr21 (A). Especially, chr10 evolves distinct original chromosome losses across the regions, termed as MSAI (B). Chromosome copy A of 10q23 and 10q24 was lost in regions R1/R3/R4, while the opposite copy B was lost in region R2, but in region R5 the whole chromosome of origin B was lost (C).

Supplementary Figure 13

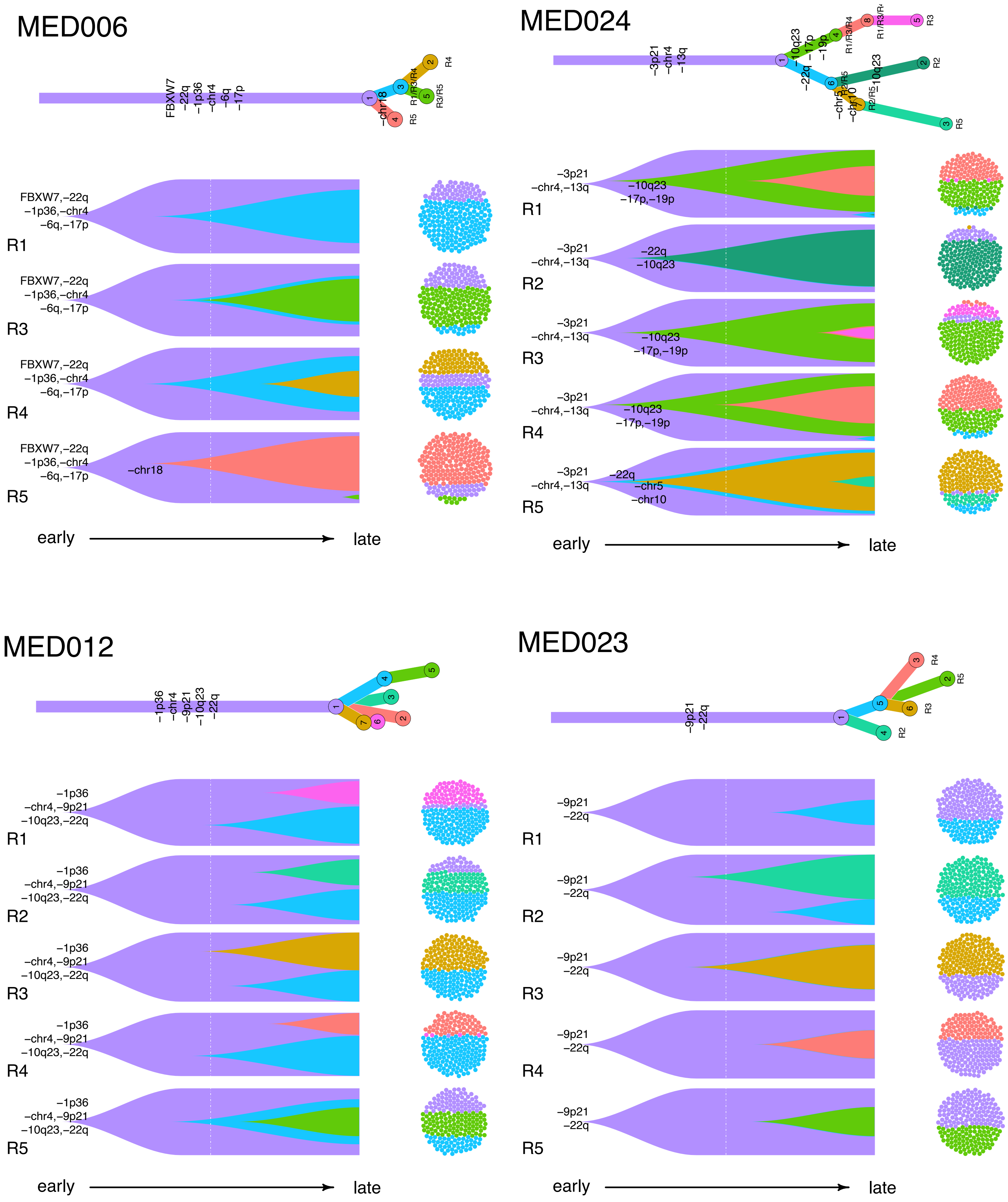

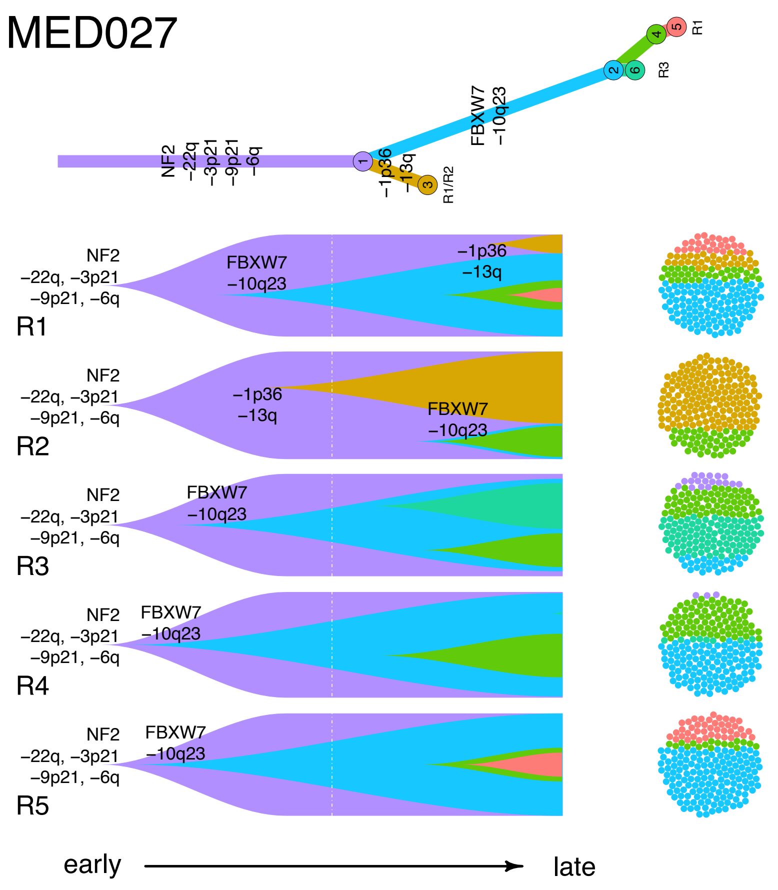
MED027
NF2
−22q
−3p21
−9p21
−6q
1
−1p36
−13q
3
R1/R2
FBXW7
−10q23
2
6
R3
4
5
R1
NF2
−22q, −3p21
−9p21, −6q
R1
FBXW7
−10q23
−1p36
−13q
R2
−1p36
−13q
FBXW7
−10q23
R3
FBXW7
−10q23
R4
FBXW7
−10q23
R5
FBXW7
−10q23
early
late

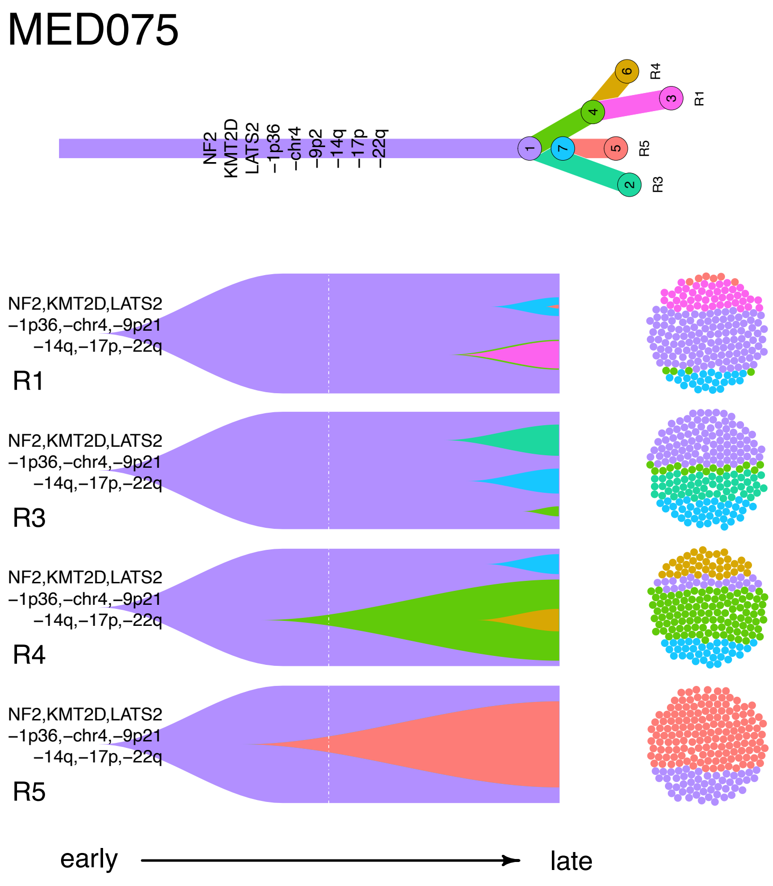
MED075
NF2
KMT2D
LATS2
−1p36
−chr4
−9p2
−14q
−17p
−22q
1
7
5
R5
2
R3
4
6
R4
3
R1
NF2,KMT2D,LATS2
−1p36,−chr4,−9p21
−14q,−17p,−22q
R1
R3
R4
R5
early
late

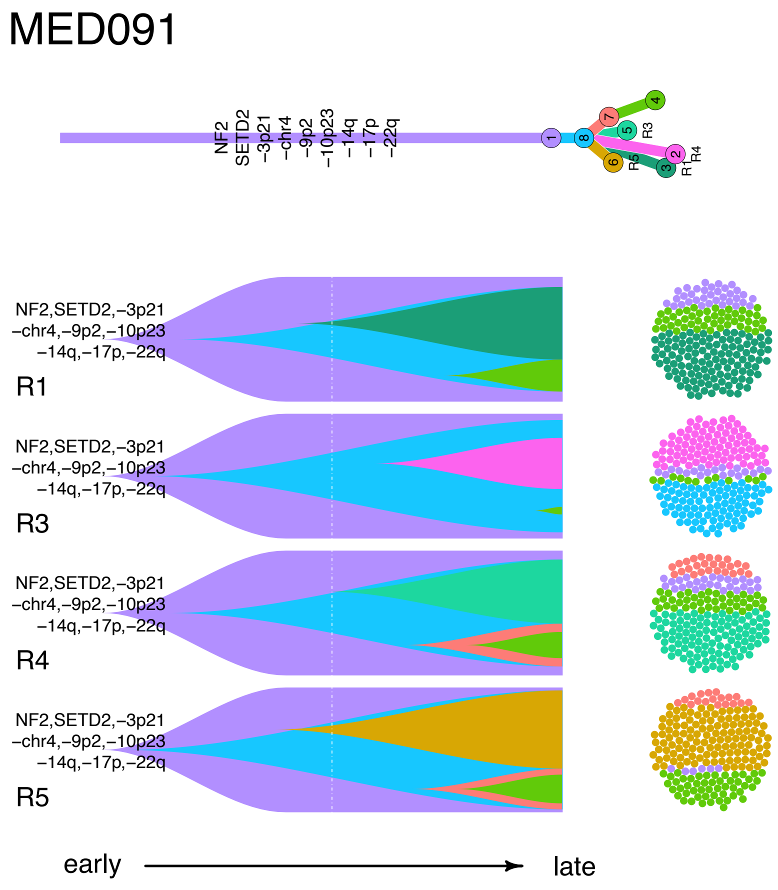
MED091
NF2
SETD2
−3p21
−chr4
−9p2
−10p23
−14q
−17p
−22q
1
8
7
4
5
R3
2
R4
6
R5
3
R1
NF2,SETD2,−3p21
−chr4,−9p2,−10p23
−14q,−17p,−22q
R1
R3
R4
R5
early
late

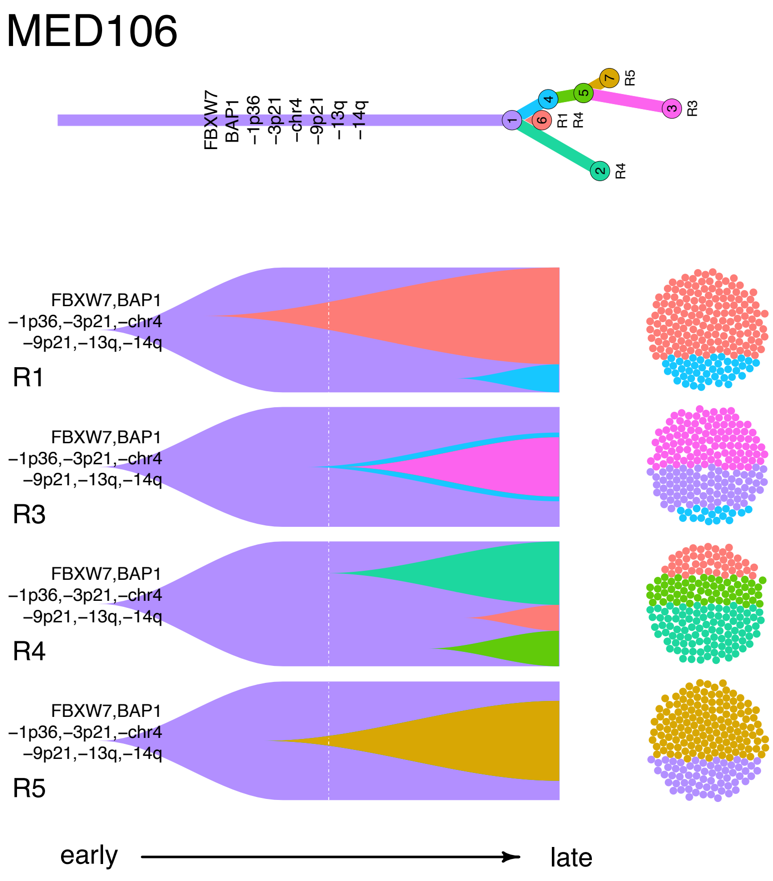
MED106
FBXW7
BAP1
−1p36
−3p21
−chr4
−9p21
−13q
−14q
1
4
5
7
R5
3
R3
6
R1
R4
2
R4
FBXW7,BAP1
−1p36,−3p21,−chr4
−9p21,−13q,−14q
R1
R3
R4
R5
early
late

Supplementary Figure 13. Subclonal evolution in MPM

Branched evolution in MEDUSA revealed by clonal deconvolution corresponding to MPMs MED006, MED024, MED012, MED023, MED027, MED075, MED091 and MED106 are represented. For each patient evolution is shown as three representations

I) a phylogenetic tree. In these trees, branch lengths are proportional to the number of substitutions in the corresponding subclone. Driver events related to each cluster are indicated on the corresponding branch, clones restricted to any regions are also labelled beside the nodes.

II) clonal evolution diagram. These Fish plot show the inferred clonal evolution over time and the proportion of cell populations for each region. Driver events belonged to each cluster are shown at the start point of the clonal expansion. A white dashed line, refers to the CCF value equal to 0.5. MED023, MED027, MED075, MED091 and MED106, consisted of subclonal truncal mutations, with high subclonal branched evolution.

III) a sphere of cells. The sphere of 200 cells (on the right for each patient) shows cell mixtures of each region. MED024 and MED027 contains subclonal driver events, which both exhibit high ITH of mutation and SCNA. While driver events of MED024 are all SCNAs, drivers of MED027 comprised both mutations and SCNAs.

Supplementary Figure 14

A

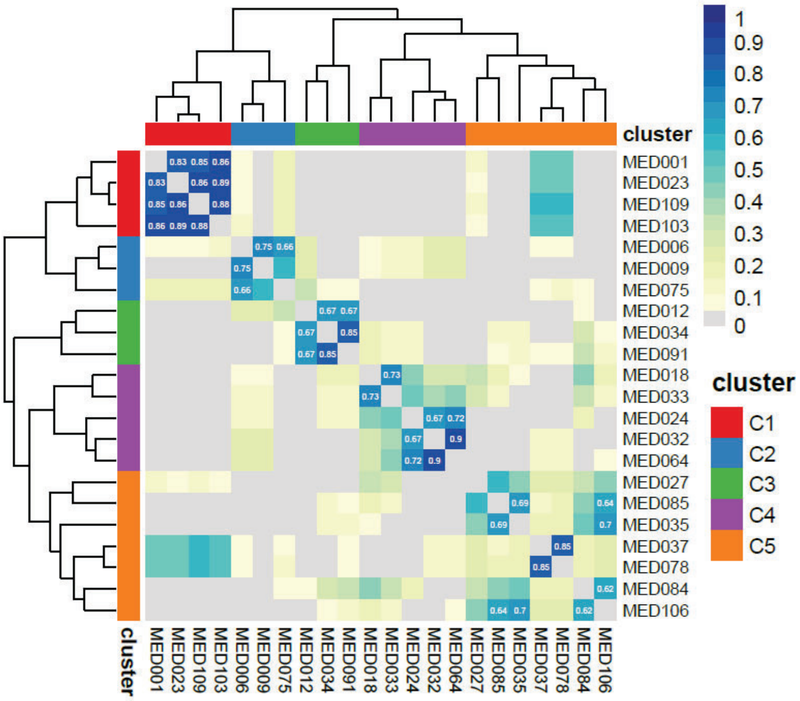

B

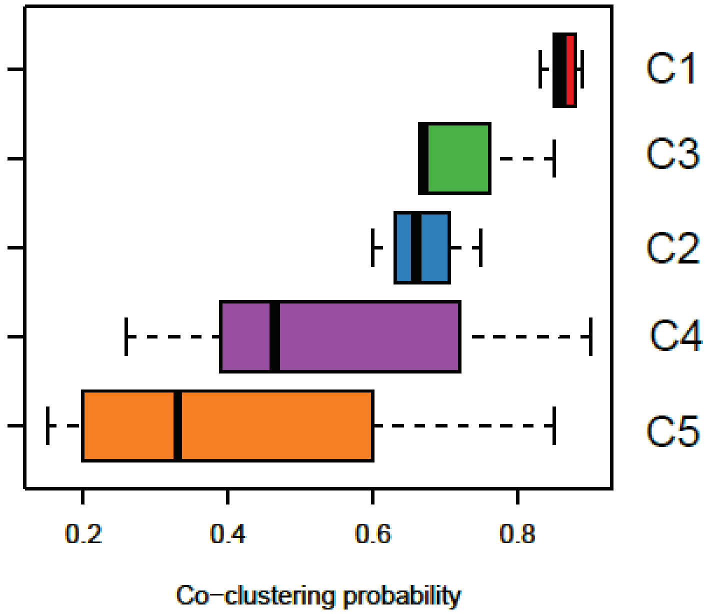

C

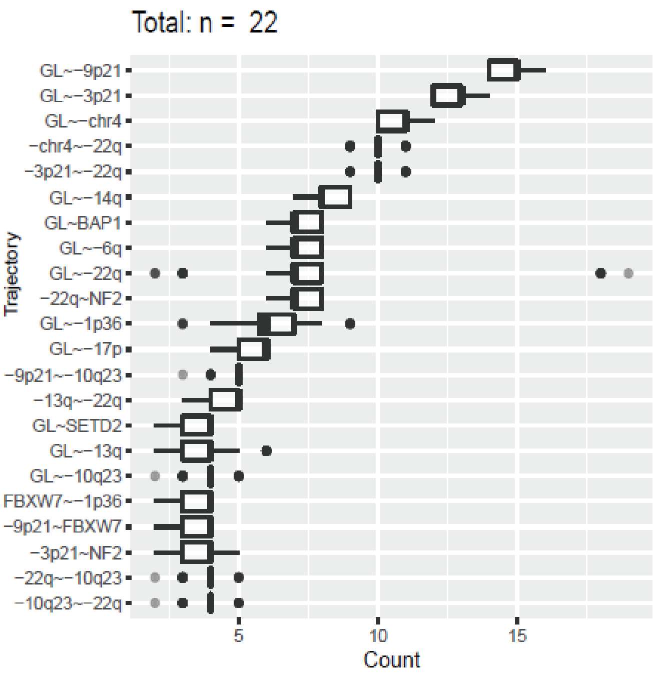

Supplementary Figure 14. Evolutionary trajectory stability

(A) we employed REVOLVER's jacknife approach to estimate evolutionary clustering stability as measured by the probability that two patients are clustered together in a resampling (N=1000) process each time removing a random percentage of patients (*p*=10%) and recomputing the fit and clustering using the original parameters. The heatmap shows the empirical probability estimated via the jacknife approach with the number of patients harbouring an edge shown across all resamples.

(B) A box plot showing the empirical probability determined using the jacknife approach but computed per cluster. The number of points used corresponds to n(n-1)/2 where n is the cluster size.

(C) Boxplot showing the number of edges per patient showing the mean and box limits corresponding to the upper (75th) and lower (25%) percentiles. The whiskers correspond to 1.5 IQR (ie. the interquartile range). Dots are outliers which are more than the 75th percentile + 1.5IQR or less than the 25% percentile -1.5 IQR.

Supplementary Figure 15

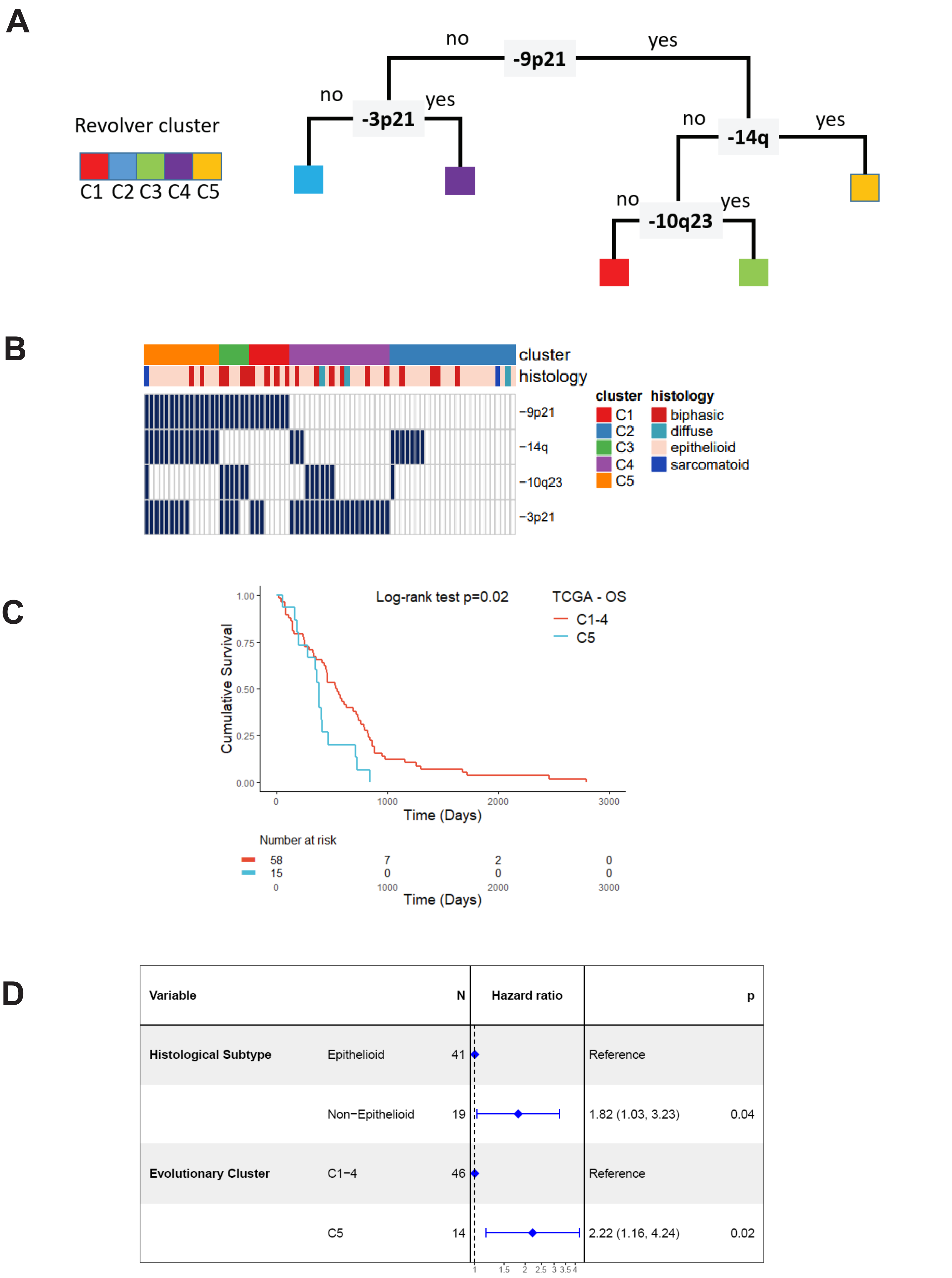

Supplementary Figure 15. Validation of C5 and prognosis in the TCGA.

A) Decision tree based filtering on evolutionary clusters in the mesothelioma TCGA. Manual decision tree generation involved filtering using the trajectory specific SCNAs -9p21, -3p21, -14q, and -10q23 to bin the independent dataset into evolutionary clusters 1 to 5

B) Heatmap summarizing the cluster c5 versus other clusters, showing exclusively epithelioid MPMs.

C. Kaplan Meier plot showing shorter overall survival for MPMs classified into the C5 evolutionary cluster in the TCGA cohort (Two-sided Log-rank test p=0.02; hazard ratio 2.01; 95% confidence intervals 1.10, 3.65). n=73 patients, the total for whom overall survival data are available.

D. Forest plot of a bivariate Cox regression model adjusting for evolutionary cluster and histology, both covariates are significantly and negatively prognostic with hazard ratio 2.22 for cluster C5 (95% confidence intervals 1.16, 4.24, Wald test p=0.04) and 1.82 for non-epithelioid histology (95% confidence intervals 1.03, 3.23, Wald test p=0.02). Global p=0.018 (Likelihood ratio test). Error bars represent 95% confidence intervals. This Cox regression model satisfied the proportionality of hazards assumption according to Schoenfeld's residuals test, with neither the individual covariates nor the overall model registering a p<0.05. All statistical tests were two-sided. n=60 patients. Only patients with the full complement of relevant data were included.

Supplementary Figure 16

A

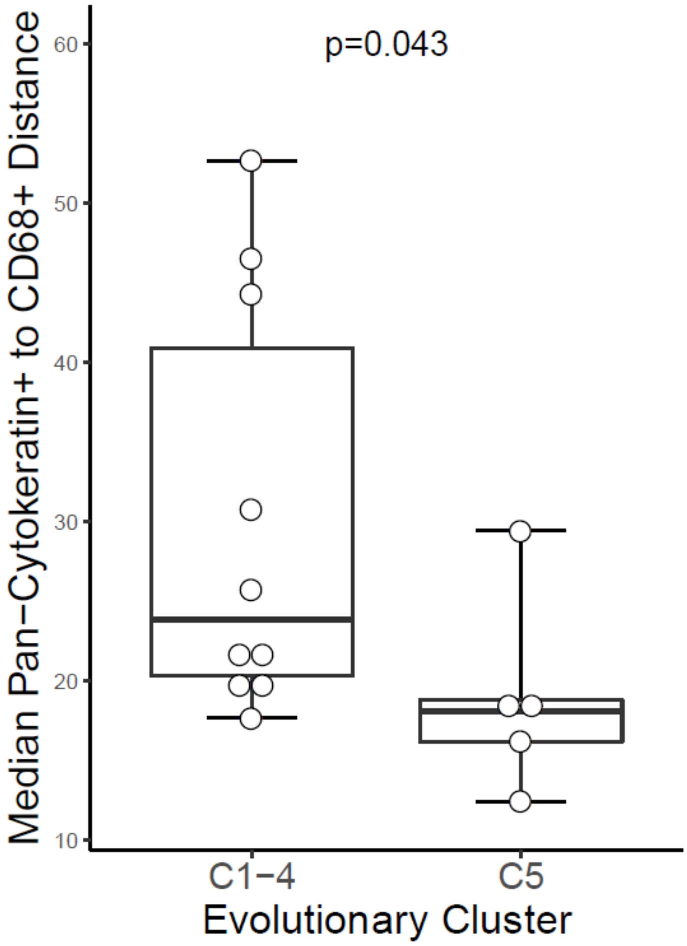

B

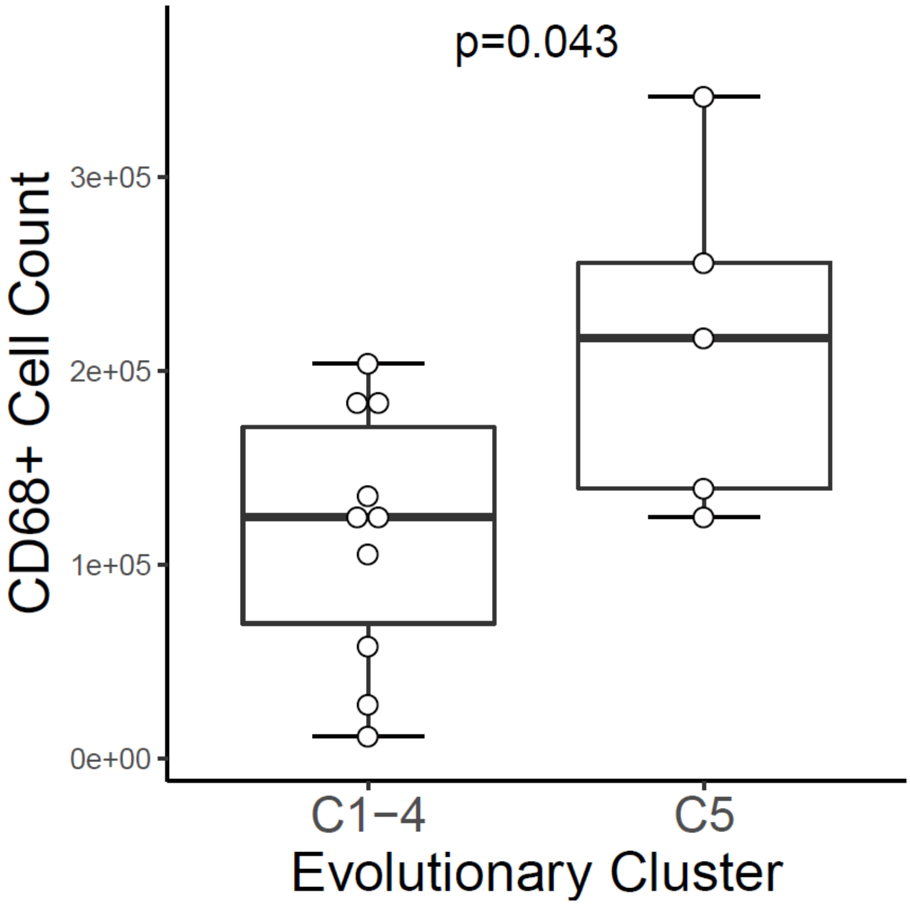

Supplementary Figure 16. Evolutionary trajectories and monocyte infiltration

A. Box plot showing that evolutionary cluster C5 MPMs exhibit deeper infiltration of $CD68^+$ myeloid cells, represented by the lower median Pan-Cytokeratin to $CD68^+$ distance, derived from spatial analysis of multiplex immunofluorescence. Two-sided Mann-Whitney U test p=0.043. n=15 patients.

B. Box plot showing higher $CD68^+$ infiltration in cluster C5 tumours, determined using quantitative multiplex immunofluorescence. Two-sided Mann-Whitney U test p=0.043. n=15 patients. Both box plots denote medians (centre lines), 25th and 75th percentiles (bounds of boxes), and minimum and maximum (whiskers).

Supplementary Figure 17

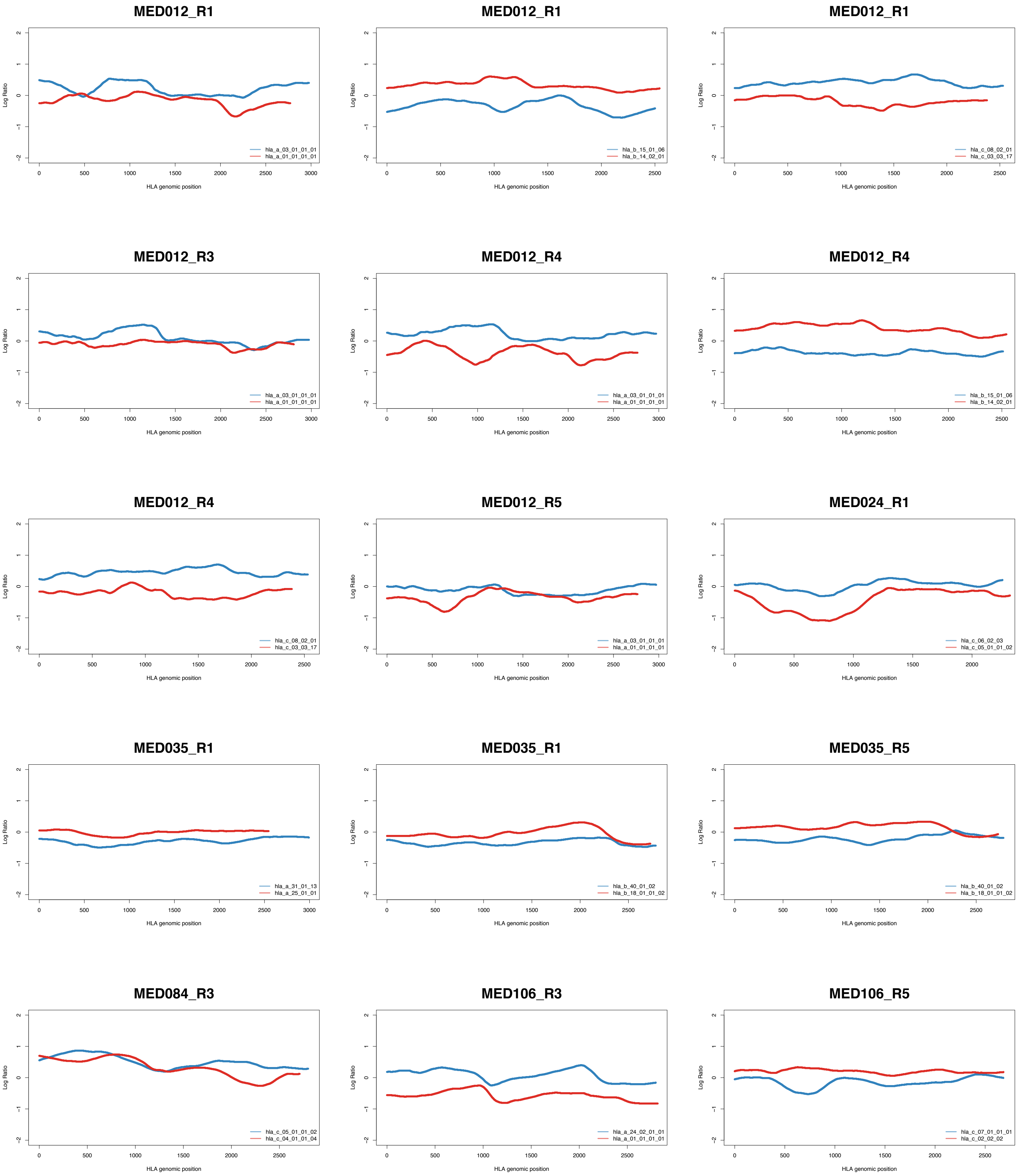

Supplementary Figure 17. Immune escape in MPM

Allele-specific HLA loss (HLA LOH) events. In the MEDUSA22 cohort, 17 HLA LOH events were detected, referring to all HLA class I genes (HLA-A, HLA-B, HLA-C). For each separated figure, x-axis represents HLA genomic position of corresponding gene, which can be seen in the bottom right with two alleles form HLA-typing result. Y-axis means adjusted log2 ratio of read depth between tumour and normal samples for each allele.
