# Supplementary material for: Clonal architecture in mesothelioma is prognostic and shapes the tumour microenvironment
Source: Nat Commun. 2021 Mar 19;12:1751. doi: 10.1038/s41467-021-21798-w (PMC7979861; doi:10.1038/s41467-021-21798-w)
Supplement: Supplementary file 3 — Description of Additional Supplementary Files [file 41467_2021_21798_MOESM3_ESM.pdf]

Description of Additional Supplementary Files

File Name: Supplementary Data 1
Description: Clinical characteristics

File Name: Supplementary Data 2
Description: Sequencing depth and coverage

File Name: Supplementary Data 3
Description: Tumor purities and ploidies estimated

File Name: Supplementary Data 4
Description: Mutational burden and intertumor heterogeneity (mutations and SCNAs)

File Name: Supplementary Data 5
Description: Evolution models in MPMs

File Name: Supplementary Data 6
Description: dN/dS ratio across cohort

File Name: Supplementary Data 7
Description: Driver gene selectivity

File Name: Supplementary Data 8
Description: Per region segmented copy number data

File Name: Supplementary Data 9
Description: Clonal mutations detected in plasma circulating total DNA

File Name: Supplementary Data 10
Description: Variant allele frequencies (VAFs) per tumour region and circulating free DNA

File Name: Supplementary Data 11
Description: Primers used for clonal mutation detection in plasma

File Name: Supplementary Data 12
Description: High frequency copy number gains and losses

File Name: Supplementary Data 13
Description: Clonal versus subclonal copy number alterations with gene annotations

File Name: Supplementary Data 14
Description: Chromosome arm-level gains or losses

File Name: Supplementary Data 15
Description: UPD segmented data

File Name: Supplementary Data 16
Description: TCGA meso data clusters C1-C5

File Name: Supplementary Data 17
Description: Neoantigens predicted.

File Name: Supplementary Data 18
Description: Loss of heterozygosity in human leukocyte antigen (HLA LOH)
