# Supplementary material for: Clonal architecture in mesothelioma is prognostic and shapes the tumour microenvironment
Source: Nat Commun. 2021 Mar 19;12:1751. doi: 10.1038/s41467-021-21798-w (PMC7979861; doi:10.1038/s41467-021-21798-w)
Supplement: Supplementary file 5 — Reporting Summary [file 41467_2021_21798_MOESM5_ESM.pdf]

nature research

Corresponding author(s): Professor Dean A. Fennell

Last updated by author(s): Jan 08 2021

Reporting Summary

Nature Research wishes to improve the reproducibility of the work that we publish. This form provides structure for consistency and transparency in reporting. For further information on Nature Research policies, see our Editorial Policies and the Editorial Policy Checklist.

Statistics

For all statistical analyses, confirm that the following items are present in the figure legend, table legend, main text, or Methods section.

n/a	Confirmed	
[ ]	[x]	The exact sample size (*n*) for each experimental group/condition, given as a discrete number and unit of measurement
[x]	[ ]	A statement on whether measurements were taken from distinct samples or whether the same sample was measured repeatedly
[ ]	[x]	The statistical test(s) used AND whether they are one- or two-sided<br>*Only common tests should be described solely by name; describe more complex techniques in the Methods section.*
[ ]	[x]	A description of all covariates tested
[ ]	[x]	A description of any assumptions or corrections, such as tests of normality and adjustment for multiple comparisons
[ ]	[x]	A full description of the statistical parameters including central tendency (e.g. means) or other basic estimates (e.g. regression coefficient) AND variation (e.g. standard deviation) or associated estimates of uncertainty (e.g. confidence intervals)
[ ]	[x]	For null hypothesis testing, the test statistic (e.g. *F*, *t*, *r*) with confidence intervals, effect sizes, degrees of freedom and *P* value noted<br>*Give P values as exact values whenever suitable.*
[x]	[ ]	For Bayesian analysis, information on the choice of priors and Markov chain Monte Carlo settings
[x]	[ ]	For hierarchical and complex designs, identification of the appropriate level for tests and full reporting of outcomes
[x]	[ ]	Estimates of effect sizes (e.g. Cohen's *d*, Pearson's *r*), indicating how they were calculated

Our web collection on statistics for biologists contains articles on many of the points above.

Software and code

Policy information about availability of computer code

Data collection	Microsoft excel 365MSO (16.0.426.30208) 64 bit
Data analysis	Due to space limitations a comprehensive list of software used in this manuscript can be found on page 8 (Appendix 1)

For manuscripts utilizing custom algorithms or software that are central to the research but not yet described in published literature, software must be made available to editors and reviewers. We strongly encourage code deposition in a community repository (e.g. GitHub). See the Nature Research guidelines for submitting code & software for further information.

Data

Policy information about availability of data

All manuscripts must include a data availability statement. This statement should provide the following information, where applicable:

- Accession codes, unique identifiers, or web links for publicly available datasets
- A list of figures that have associated raw data
- A description of any restrictions on data availability

A full data availability statement and code availability statement has been included in the revised manuscript

The main data supporting the results are available within this article and its supplementary information.
Sequence data have been deposited at the BioProject Archive [https://www.ncbi.nlm.nih.gov/bioproject/PRJNA649889], which is hosted by the national Centre for Biotechnology Information, under accession number PRJNA649889. Scripts related to clonality analysis, mutational signatures, dn/dS analysis and REVOLVER have been deposited in Github. All of the other data supporting the findings of this study are available within the article and its supplementary information files and from the corresponding author upon reasonable request.

Field-specific reporting

Please select the one below that is the best fit for your research. If you are not sure, read the appropriate sections before making your selection.

- [x] Life sciences
- [ ] Behavioural & social sciences
- [ ] Ecological, evolutionary & environmental sciences

For a reference copy of the document with all sections, see nature.com/documents/nr-reporting-summary-flat.pdf

Life sciences study design

All studies must disclose on these points even when the disclosure is negative.

Sample size	Patients were deemed eligible for this study if they had resectable malignant pleural mesothelioma and were scheduled to undergo extended pleurectomy decortication. sample size was determined by a number of factors, including patient eligibility, quality control of DNA, filtering criteria related to exome sequencing analysis. Based on the size of the cohort, the statistically significant results presented are hypothesis generating for larger studies with pre-determined sample size
Data exclusions	No data was excluded
Replication	Experiments were completed in triplicate to ensure reproducibility. Multiregional whole exome sequencing was was conducted in patients using repeated tissue sampling (4-5 regions)
Randomization	The study was non-randomized. Repeated evolution was investigated using transfer learning
Blinding	Bioinformaticians were blinded to the clinical histories related to the patient cohort

Behavioural & social sciences study design

All studies must disclose on these points even when the disclosure is negative.

Study description	
Research sample	
Sampling strategy	
Data collection	
Timing	
Data exclusions	
Non-participation	
Randomization	

Ecological, evolutionary & environmental sciences study design

All studies must disclose on these points even when the disclosure is negative.

Study description	*Briefly describe the study. For quantitative data include treatment factors and interactions, design structure (e.g. factorial, nested, hierarchical), nature and number of experimental units and replicates.*

Research sample	*Describe the research sample (e.g. a group of tagged Passer domesticus, all Stenocereus thurberi within Organ Pipe Cactus National Monument), and provide a rationale for the sample choice. When relevant, describe the organism taxa, source, sex, age range and any manipulations. State what population the sample is meant to represent when applicable. For studies involving existing datasets, describe the data and its source.*
Sampling strategy	*Note the sampling procedure. Describe the statistical methods that were used to predetermine sample size OR if no sample-size calculation was performed, describe how sample sizes were chosen and provide a rationale for why these sample sizes are sufficient.*
Data collection	*Describe the data collection procedure, including who recorded the data and how.*
Timing and spatial scale	*Indicate the start and stop dates of data collection, noting the frequency and periodicity of sampling and providing a rationale for these choices. If there is a gap between collection periods, state the dates for each sample cohort. Specify the spatial scale from which the data are taken*
Data exclusions	*If no data were excluded from the analyses, state so OR if data were excluded, describe the exclusions and the rationale behind them, indicating whether exclusion criteria were pre-established.*
Reproducibility	*Describe the measures taken to verify the reproducibility of experimental findings. For each experiment, note whether any attempts to repeat the experiment failed OR state that all attempts to repeat the experiment were successful.*
Randomization	*Describe how samples/organisms/participants were allocated into groups. If allocation was not random, describe how covariates were controlled. If this is not relevant to your study, explain why.*
Blinding	*Describe the extent of blinding used during data acquisition and analysis. If blinding was not possible, describe why OR explain why blinding was not relevant to your study.*

Did the study involve field work? ☐ Yes ☐ No

Field work, collection and transport

Field conditions	*Describe the study conditions for field work, providing relevant parameters (e.g. temperature, rainfall).*
Location	*State the location of the sampling or experiment, providing relevant parameters (e.g. latitude and longitude, elevation, water depth).*
Access & import/export	*Describe the efforts you have made to access habitats and to collect and import/export your samples in a responsible manner and in compliance with local, national and international laws, noting any permits that were obtained (give the name of the issuing authority, the date of issue, and any identifying information).*
Disturbance	*Describe any disturbance caused by the study and how it was minimized.*

Reporting for specific materials, systems and methods

We require information from authors about some types of materials, experimental systems and methods used in many studies. Here, indicate whether each material, system or method listed is relevant to your study. If you are not sure if a list item applies to your research, read the appropriate section before selecting a response.

Materials & experimental systems

n/a	Involved in the study	
☐	☑	Antibodies
☐	☑	Eukaryotic cell lines
☒	☐	Palaeontology and archaeology
☒	☐	Animals and other organisms
☐	☑	Human research participants
☐	☑	Clinical data
☒	☐	Dual use research of concern

Methods

n/a	Involved in the study	
☒	☐	ChIP-seq
☐	☒	Flow cytometry
☒	☐	MRI-based neuroimaging

Antibodies

Antibodies used	Due to space limitations a comprehensive list of the antibodies used in this manuscript can be found on page 9 (Appendix 2)
Validation	Anti-YAP subcellular location was used to validate the antibody in the context of whole exome sequenced cell lines harbouring wild type or mutant NF2<br>All antibodies were commercial and validated by the manufacturer

Eukaryotic cell lines

Policy information about cell lines

Cell line source(s)	Cell lines were generated from patient's tumours (ie. primary cell lines)
Authentication	Primary cell lines were whole exome sequenced and somatic mutations verified relative to the primary tumour
Mycoplasma contamination	Cell lines were confirmed negative for contamination with mycoplasma
Commonly misidentified lines (See ICLAC register)	Not applicable. only MEDUSA cell lines derived from patients tumours in this cohort were used

Palaeontology and Archaeology

Specimen provenance	*Provide provenance information for specimens and describe permits that were obtained for the work (including the name of the issuing authority, the date of issue, and any identifying information).*
Specimen deposition	*Indicate where the specimens have been deposited to permit free access by other researchers.*
Dating methods	*If new dates are provided, describe how they were obtained (e.g. collection, storage, sample pretreatment and measurement), where they were obtained (i.e. lab name), the calibration program and the protocol for quality assurance OR state that no new dates are provided.*

☐ Tick this box to confirm that the raw and calibrated dates are available in the paper or in Supplementary Information.

Ethics oversight	*Identify the organization(s) that approved or provided guidance on the study protocol, OR state that no ethical approval or guidance was required and explain why not.*

Note that full information on the approval of the study protocol must also be provided in the manuscript.

Animals and other organisms

Policy information about studies involving animals; ARRIVE guidelines recommended for reporting animal research

Laboratory animals	*For laboratory animals, report species, strain, sex and age OR state that the study did not involve laboratory animals.*
Wild animals	*Provide details on animals observed in or captured in the field; report species, sex and age where possible. Describe how animals were caught and transported and what happened to captive animals after the study (if killed, explain why and describe method; if released, say where and when) OR state that the study did not involve wild animals.*
Field-collected samples	*For laboratory work with field-collected samples, describe all relevant parameters such as housing, maintenance, temperature, photoperiod and end-of-experiment protocol OR state that the study did not involve samples collected from the field.*
Ethics oversight	*Identify the organization(s) that approved or provided guidance on the study protocol, OR state that no ethical approval or guidance was required and explain why not.*

Note that full information on the approval of the study protocol must also be provided in the manuscript.

Human research participants

Policy information about studies involving human research participants

Population characteristics	Patients had early stage malignant pleural mesothelioma that was deemed resectable, and were planned for routine extended pleurectomy decortication Patients were aged over 18, could be male or female and required a confirmed histological diagnosis of mesothelioma
Recruitment	Patients were considered eligible for tissue collection if they met the population characteristics summarised above
Ethics oversight	The study was approved by a National ethics committee IRAS ID 131283

Clinical data

Policy information about clinical studies

All manuscripts should comply with the ICMJEguidelines for publication of clinical research and a completedCONSORT checklist must be included with all submissions.

Clinical trial registration	NIHR portfolio study 17674, IRAS ID 131283, MREC ID, 14/LO/1527

Describe the covariate-relevant population characteristics of the human research participants (e.g. age, gender, genotypic information, past and current diagnosis and treatment categories). If you filled out the behavioural & social sciences study design questions and have nothing to add here, write "See above."

Describe how participants were recruited. Outline any potential self-selection bias or other biases that may be present and how these are likely to impact results.

Study protocol	A clinical trial protocol is not available. MEDUSA is a non-interventional/observational, retrospective pilot cohort study
Data collection	Clinical data was collected within a single UK based NHS Trust (university Hospitals of Leicester NHS Trust) between November 2014 and May 2018
Outcomes	Progression free survival is defined as the time of surgical resection until radiological disease progression. Overall survival is defined as the time from surgical resection until death

Dual use research of concern

Policy information about dual use research of concern

Hazards

Could the accidental, deliberate or reckless misuse of agents or technologies generated in the work, or the application of information presented in the manuscript, pose a threat to:

No	Yes	
X	☐	Public health
X	☐	National security
X	☐	Crops and/or livestock
X	☐	Ecosystems
X	☐	Any other significant area

Experiments of concern

Does the work involve any of these experiments of concern:

No	Yes	
X	☐	Demonstrate how to render a vaccine ineffective
X	☐	Confer resistance to therapeutically useful antibiotics or antiviral agents
X	☐	Enhance the virulence of a pathogen or render a nonpathogen virulent
X	☐	Increase transmissibility of a pathogen
X	☐	Alter the host range of a pathogen
X	☐	Enable evasion of diagnostic/detection modalities
X	☐	Enable the weaponization of a biological agent or toxin
X	☐	Any other potentially harmful combination of experiments and agents

ChIP-seq

Data deposition

☐ Confirm that both raw and final processed data have been deposited in a public database such as GEO.

☐ Confirm that you have deposited or provided access to graph files (e.g. BED files) for the called peaks.

Data access links<br>*May remain private before publication.*	*For "Initial submission" or "Revised version" documents, provide reviewer access links. For your "Final submission" document, provide a link to the deposited data.*
Files in database submission	*Provide a list of all files available in the database submission.*
Genome browser session<br>(e.g. UCSC)	*Provide a link to an anonymized genome browser session for "Initial submission" and "Revised version" documents only, to enable peer review. Write "no longer applicable" for "Final submission" documents.*

Methodology

Replicates	*Describe the experimental replicates, specifying number, type and replicate agreement.*
Sequencing depth	*Describe the sequencing depth for each experiment, providing the total number of reads, uniquely mapped reads, length of reads and whether they were paired- or single-end.*
Antibodies	*Describe the antibodies used for the ChIP-seq experiments; as applicable, provide supplier name, catalog number, clone name, and lot number.*
Peak calling parameters	*Specify the command line program and parameters used for read mapping and peak calling, including the ChIP, control and index files used.*

Data quality: *Describe the methods used to ensure data quality in full detail, including how many peaks are at FDR 5% and above 5-fold enrichment.*

Software: *Describe the software used to collect and analyze the ChIP-seq data. For custom code that has been deposited into a community repository, provide accession details.*

Flow Cytometry

Plots

Confirm that:

- ☐ The axis labels state the marker and fluorochrome used (e.g. CD4-FITC).
- ☐ The axis scales are clearly visible. Include numbers along axes only for bottom left plot of group (a 'group' is an analysis of identical markers).
- ☐ All plots are contour plots with outliers or pseudocolor plots.
- ☐ A numerical value for number of cells or percentage (with statistics) is provided.

Methodology

Sample preparation: Cells have been resuspended in PBS (10209252, Oxoid) with 0.5% of BSA (A7030, Sigma-Aldrich) and aliquoted 1x105 per tube.Prepared aliquots have been incubated for 30 minutes at 4OC with one or both of fluorophore-conjugated antibody or used as unstained control. Dilution of both antibodies was 1:10. After incubation samples were washed 3 times in PBS with 0.5% BSA (5 minutes at 300xG) and resuspended to final volume of 300 µl. Samples have been acquired on flow cytometer directly after preparation.

Instrument: BD FACS CantoII

Software: BD FACSDiva8.0.1

Cell population abundance: MED85 7500 cells with 25.9% positive for mesothelin and 97.7% positive for podoplanin.
MED96 10000 cells with 17.4% positive for mesothelin and 55.7% positive for podoplanin.

Gating strategy: Samples were acquired first on FSC vs SSC and generous gate have been created.
Threshold for APC and PE channels have been set on unstained controls.

☐ Tick this box to confirm that a figure exemplifying the gating strategy is provided in the Supplementary Information.

Magnetic resonance imaging

Experimental design

Design type: *Indicate task or resting state; event-related or block design.*

Design specifications: *Specify the number of blocks, trials or experimental units per session and/or subject, and specify the length of each trial or block (if trials are blocked) and interval between trials.*

Behavioral performance measures: *State number and/or type of variables recorded (e.g. correct button press, response time) and what statistics were used to establish that the subjects were performing the task as expected (e.g. mean, range, and/or standard deviation across subjects).*

Acquisition

Imaging type(s): *Specify: functional, structural, diffusion, perfusion.*

Field strength: *Specify in Tesla*

Sequence & imaging parameters: *Specify the pulse sequence type (gradient echo, spin echo, etc.), imaging type (EPI, spiral, etc.), field of view, matrix size, slice thickness, orientation and TE/TR/flip angle.*

Area of acquisition: *State whether a whole brain scan was used OR define the area of acquisition, describing how the region was determined.*

Diffusion MRI ☐ Used ☐ Not used

Preprocessing

Preprocessing software: *Provide detail on software version and revision number and on specific parameters (model/functions, brain extraction, segmentation, smoothing kernel size, etc.).*

Normalization: *If data were normalized/standardized, describe the approach(es): specify linear or non-linear and define image types used for transformation OR indicate that data were not normalized and explain rationale for lack of normalization.*

Normalization template | *Describe the template used for normalization/transformation, specifying subject space or group standardized space (e.g. original Talairach, MNI305, ICBM152) OR indicate that the data were not normalized.*

Noise and artifact removal | *Describe your procedure(s) for artifact and structured noise removal, specifying motion parameters, tissue signals and physiological signals (heart rate, respiration).*

Volume censoring | *Define your software and/or method and criteria for volume censoring, and state the extent of such censoring.*

Statistical modeling & inference

Model type and settings | *Specify type (mass univariate, multivariate, RSA, predictive, etc.) and describe essential details of the model at the first and second levels (e.g. fixed, random or mixed effects; drift or auto-correlation).*

Effect(s) tested | *Define precise effect in terms of the task or stimulus conditions instead of psychological concepts and indicate whether ANOVA or factorial designs were used.*

Specify type of analysis: ☐ Whole brain ☐ ROI-based ☐ Both

Statistic type for inference (See Eklund et al. 2016) | *Specify voxel-wise or cluster-wise and report all relevant parameters for cluster-wise methods.*

Correction | *Describe the type of correction and how it is obtained for multiple comparisons (e.g. FWE, FDR, permutation or Monte Carlo).*

Models & analysis

n/a	Involved in the study
☐	☐ Functional and/or effective connectivity
☐	☐ Graph analysis
☐	☐ Multivariate modeling or predictive analysis

Functional and/or effective connectivity | *Report the measures of dependence used and the model details (e.g. Pearson correlation, partial correlation, mutual information).*

Graph analysis | *Report the dependent variable and connectivity measure, specifying weighted graph or binarized graph, subject- or group-level, and the global and/or node summaries used (e.g. clustering coefficient, efficiency, etc.).*

Multivariate modeling and predictive analysis | *Specify independent variables, features extraction and dimension reduction, model, training and evaluation metrics.*

Appendix 1 - Software

- Burrows-Wheeler Aligner (bwa-0.7.17)
- Sambamba (v0.6.7)
- Picard tools (v2.18.9)
- SAMtools (v1.8)
- VarScan2 somatic (v2.3)
- MuTect2
- SAMtools mpileup (1.0)
- GATK bundle (4.0.5.1)
- Annovar (14 Dec 2015)
- SIFT,
- Polyphen v2
- CScape v1
- MutationTaster
- ContEst (1.0)
- Absolute
- Ascat
- CNVkit
- Battenberg
- CITUP, v0.1.1
- Conevol
- Revolver R version 3.5.1
- dNdScv.0.1.0
- deconstructSigs
- Polysolver (v1.0)
- MutsigCV(v1.4)
- LOHHLA v1
- pVACseq(4.0.9)
- Bam-readcount (0.8.0)
- NetMHC
- Variant Effect Predictor (Version 84)
- NetMHCpan
- pVACseq toolkit
- CIBERSORT Jar Version 1.06 (May 5, 2017)
- R Studio version 1.1.463
- Phenochart inForm® (Akoya Biosciences)
- ImageJ software (NIH)
- Primer3 tool (Whitehead Institute, MIT)
- OligoAnalyzer 3.1 (Integrated DNA Technologies)
- QuantaSoft™ Software v1.7 (Bio-Rad)
- R Studio, version 1.2.5001, running R version 3.6.1
- GraphPad Prism 7 (GraphPad Prism Software Inc., CA, USA)

Appendix 2 - Antibodies

- Material name - anti- human mesothelin – PE conjugated; Manufacturer – R&D systems; Product code- 32652P, lot number: AANW0315081
- Material name - anti- podoplanin – APC conjugated, Manufacturer – Miltenyi biotec; Product code- 130-107-016, lot number: 5180129533
- Material name - anti-YAP1 antibody Manufacturer – Santa Cruz Biotechnology, Product code- sc- 101199
- Material name - chicken anti-mouse IgG (H+L)-AlexaFluor488 32652P Product code- 1597055, Manufacturer – Thermo Fisher Scientific
- Material name - CD68, clone KP1, Manufacturer – Dako, Product code - M0814, lot number 20047711
- Material name - CD8, clone C8/144B , Manufacturer – Dako, Product code - M7103, lot number 20078591
- Material name - Pan-cytokeratin, clone MNF116 Manufacturer – Dako, Product code - M0821, Lot number 20078396
- Material name - Opal™ Polymer HRP-conjugated secondary antibody Ms + Rb, Manufacturer – Perkin Elmer, Product code - ARH1001 lot number 191212018
